# MOSIC: Model-Agnostic Optimal Subgroup Identification with Multi-Constraint for Improved Reliability

**DOI:** 10.1145/3770855.3818926

**Published:** 2026-08-08

**Authors:** Wenxin Chen, Weishen Pan, Kyra Gan, Fei Wang

**Affiliations:** Computer Science, Cornell University, New York City, New York, USA; Weill Cornell Medicine, Cornell University, New York City, New York, USA; Operations Research and Information Engineering, Cornell University, New York City, New York, USA; Weill Cornell Medicine, Cornell University, New York City, New York, USA

**Keywords:** Causal Inference, Heterogeneous Treatment Effect, Optimal Subgroup Identification, Constrained Optimization

## Abstract

Current subgroup identification methods typically follow a two-step approach: first estimate *conditional average treatment effects* (CATEs) and then apply thresholding or rule-based procedures to define subgroups. While intuitive, this decoupled approach fails to incorporate key constraints essential for real-world clinical decision-making—such as subgroup size and propensity overlap. These constraints operate on fundamentally different axes than CATE estimation and are not naturally accommodated within existing frameworks, thereby limiting the practical applicability of these methods. We propose a *unified optimization framework* that directly solves the *primal* constrained optimization problem to identify optimal subgroups. Our key innovation is a reformulation of the constrained primal problem as an *unconstrained differentiable min-max objective*, solved via a gradient descent-ascent algorithm. We theoretically establish that our solution converges to a feasible and locally optimal solution. Unlike threshold-based CATE methods that apply constraints as post-hoc filters, our approach enforces them directly during optimization. The framework is *model-agnostic*, compatible with a wide range of CATE estimators, and extensible to additional constraints like cost limits or fairness criteria. Extensive experiments on synthetic and real-world datasets demonstrate its effectiveness in identifying high-benefit subgroups while maintaining better satisfaction of constraints.

## 1 Introduction

In precision medicine, a fundamental challenge is identifying patient subgroups that benefit most from specific treatments, where heterogeneous effects must be estimated from observational data [31, 32]. Most existing methods adopt a two-step paradigm: they first estimate CATEs using machine learning methods, then derive subgroups through either thresholding or simplified rule-based models (see Section 2).

However, this two-stage approach falls short in real-world settings, where optimal subgroup identification must account for diverse, interacting constraints. Clinical deployment often requires satisfying statistical conditions like minimum subgroup size and overlap [17, 53], as well as operational and ethical considerations such as budget, safety, and fairness. Existing methods typically treat these constraints as post-hoc filters rather than integrating them into the optimization process. As a result, they *struggle to jointly satisfy multiple constraints*, leading to instability and poor performance. These challenges highlight a deeper disconnect between the continuous nature of CATE estimates and the discrete, constraint-driven structure of clinically actionable subgroup identification. This motivates the need for new frameworks that can incorporate and optimize over multiple real-world constraints in a unified and principled way.

We propose MOSIC (Model-agnostic Optimal Subgroup Identification with multi-Constraints), a *novel optimization framework* that identifies subgroups with maximal CATE while satisfying group size and overlap constraints—with flexibility to incorporate additional constraints. Our approach addresses the challenge of nonconvex/nonconcave optimization, and our contributions are threefold:

- **Problem Reformulation for Stable Solutions:** We develop a stable optimization procedure that: (1) formulates the task as a constrained problem (Section 3.1), (2) absorbs constraints into the objective via a reformulation (Section 3.2), and (3) modifies the objective to improve stability and solves it using a gradient descent-ascent algorithm (Section 4.1). Finally, we establish that the resulting solution is locally optimal and feasible (Section 4.2).
- **Flexibility:** MOSIC offers flexibility across three dimensions: (1) supporting multiple subgroup model architectures (e.g., multilayer perceptrons, decision trees) for different performance-interpretability tradeoffs, (2) compatibility with various ATE estimators, and (3) extensibility to diverse clinical constraints beyond our focus on size and overlap.
- **Comprehensive Evaluation:** We extensively evaluate our framework on both synthetic and real-world data, demonstrating its effectiveness in optimal subgroup identification under multiple constraints (Section 5). Our implementation is available at https://github.com/Wenxin-Elmon-Chen/MOSIC-KDD. Supplementary appendix is available at https://github.com/Wenxin-Elmon-Chen/MOSIC-KDD/blob/main/supplementary/appendix.pdf.

## 2 Related Work

We review treatment effect estimation, overlap handling, subgroup identification, and constrained optimization, highlighting that multi-constraint subgroup identification remains under-explored.

### Treatment Effect Estimation.

MOSIC accommodates various average treatment effect (ATE) estimators. Traditional methods like IPTW, meta-learners [33], R-learner [41], BART [14] rely on either the treatment or outcome model, making them sensitive to model misspecification. In contrast, doubly robust estimators such as AIPTW [45], DR-learner [29], TMLE [52], and DML [13] require only one model (treatment or outcome) to be correctly specified. This paper adopts AIPTW for illustration.

While ATE captures population-level effects, CATE estimation enables subgroup-specific recommendations. Modern methods include 1) tree-based methods like Causal Tree (CT) [3], Causal Forest (CF) [55], and 2) neural-network-based approaches, such as TAR-Net [47] and Dragonnet [48]. In our AIPTW estimator, we estimate the outcome model with Dragonnet and the treatment model with Logistic Regression (LR).

### Dealing with Limited Overlap.

Limited sample overlap can bias treatment effect estimates or inflate variance. Common solutions include truncating propensity scores [16, 23] and excluding low-overlap units [17, 28, 35, 46]. We adopt the latter approach by incorporating a set of constraints to avoid low-overlap regions in subgroup identification. This ensures more reliable ATE estimation within the identified subgroup.

### Optimal Subgroup Identification.

Existing methods fall into three categories: (i) baseline methods without interpretability or constraints, (ii) *interpretable* methods, and (iii) *constrained* methods.

*Baseline methods* either: (1) rank patients by estimated CATE values [11, 53] (we benchmarked this approach employing CT, CF, and Dragonnet in Section 5) and DR-learner, R-learner, BART in Appendix E.4, or (2) optimize individual treatment rules using methods like Outcome-Weighted Learning (OWL) [37, 60]. These methods provide useful benchmarks but do not readily accommodate additional constraints, a limitation that our approach addresses.

*Interpretable methods* often rely on *Decision Tree* (DT) [4, 19, 25, 36]. A representative example is Virtual Twins (VT) [20]. It first estimates CATE and then applies a DT for interpretable subgroup identification. Beyond trees, rule learning approaches have been adopted [56, 61]. However, these methods rely on combinatorial searches and do not scale. Our method can instead leverage DTs as the backbone model, achieving the same level of interpretability while remaining scalable. We compare its performance against other DT-based methods in Section 5.3.

*Constrained methods* explicitly incorporate constraints into subgroup search. CAPITAL [10] is the most closely related approach to ours: it maximizes subgroup size under a single constraint on subgroup ATE and allows extension to one additional constraint via Lagrangian relaxation. However, it struggles with multiple constraints due to instability and a lack of feasibility guarantees. In Section 5, we compare our approach with VT, OWL, and CAPITAL.

Related ideas also appear in constrained policy learning, where the goal is to optimize policies subject to explicit safety and budget constraints. Examples include constrained policy optimization [1, 43], contextual bandits with knapsacks [6, 50], and safe RL [22, 59]. These methods generally impose trajectory-level cumulative cost or risk constraints in sequential decision-making settings. In contrast, MOSIC addresses a different class of structural constraints aimed at improving the reliability of a learned subgroup rule in the static setting, such as the overlap and group-size constraints. It can additionally accommodate linear and ratio-form constraints such as risk and budget restrictions.

### Constrained Optimization.

Constrained optimization algorithms vary by problem convexity. For *convex objective and constraints*, classical methods such as Projected Gradient Descent (PGD), Frank-Wolfe (FW), Interior Point Methods (IPM), and Lagrangian-based methods (e.g., Alternating Direction Method of Multipliers, ADMM [8]) are effective. For *non-convex objectives with convex feasible regions*, global optimality is NP-hard, and convergence guarantees are typically weakened [34, 57]. Our setting—*non-convex objective with non-convex constraints*—poses greater challenges: PGD struggles with complex projection, IPM scales poorly with constraint count, FW assumes convexity, and Lagrangian methods often lack stability and feasibility guarantees. ADMM fails here due to the non-separable objective. While ADMM variants exist for structured problems [21], none apply to our setting. In contrast, our method guarantees both constraint feasibility and local optimality, critical for real-world deployment.

## 3 Problem Setting

We consider an observational dataset with baseline covariates $X\in 𝒳\subseteq R^{d}$ (e.g., severity scores, laboratory measurements, and comorbidity), binary treatment assignment A∈𝒜={0,1} (e.g., corticosteroids), and clinical outcome Y∈𝒴, where Y may be binary (e.g., mortality) or continuous. The dataset consists of n samples ${\left(x_{i},a_{i},y_{i}\right)}_{i=1}^{n}$.

A subgroup in our setting is a subset of patients defined by X, such as “patients with high inflammation biomarkers and moderate respiratory severity”. Our goal is to identify a subgroup in which the treatment effect is favorable, while ensuring the subgroup is sufficiently large and has adequate treatment-control overlap in the observed data so that the estimated ATE is reliable.

Let Y(0) and Y(1) denote the potential outcomes under control and treatment. The propensity score is denoted as e(x)=P(A=1∣X=x), and potential outcomes as $\mu_{a}(x)=E[Y|X=x,A=a]$. Throughout this work, we adopt standard causal inference assumptions: 1) Stable Unit Treatment Value Assumption (SUTVA): Y=A⋅Y(1)+(1-A)⋅Y(0); 2) Unconfoundedness: {Y(0),Y(1)}⊥A∣X; 3) Overlap: 0<e(x)<1,∀x∈𝒳.

### 3.1 Reliable Subgroup Identification Framework

We define a subgroup identification model: $S:R^{d}\mapsto \{0,1\}$, which assigns a patient with covariates X=x to the subgroup (S(x)=1) or excludes them (S(x)=0). The target estimand is subgroup ATE: E[Y(1)-Y(0)∣S(X)=1], which reads as “the mean difference of potential outcome under treatment and that under control, within the selected subgroup”. Under SUTVA and unconfoundedness, this estimand can be expressed as

$$E[E[Y|A=1,X]-E[Y|A=0,X]|\tilde{S}(X)=1].$$

Let $\overset{ˆ}{\phi }\left(x_{i},a_{i},y_{i}\right)$ denote the estimated pseudo-outcomes for each sample, and 1(⋅) as the indicator function, a general estimator of the subgroup ATE is then

$$\frac{\sum_{i=1}^{n}1(\tilde{S}(X)=1)\overset{ˆ}{\phi }\left(x_{i},a_{i},y_{i}\right)}{\sum_{i=1}^{n}1(\tilde{S}(X)=1)}.$$

Our goal is to identify a subgroup such that its subgroup ATE is maximized, while ensuring the following constraints:

- **Minimum size requirement.** A sufficiently large subgroup is essential for reliable estimation, robust statistical power, and economic viability in applications like drug repurposing.
- **Each sample in the identified subgroup lies in a region with sufficient overlap.** Intuitively, the propensity score e(x) represents the treatment assignment probability given covariates. Patients with e(x) close to 0 or 1 lie in low-overlap regions, meaning that either one of the treatment arms is rarely observed for patients with X=x, making the effect estimation and subsequent subgroup identification unreliable. For this reason, we impose that each sample in the selected subgroup has a propensity score bounded away from 0 and 1, similar to excluding samples with extreme propensity scores [17].

The above task can be formally stated as follows:

(Problem I) $$\underset{\tilde{S}}{\text{min}}-\frac{\sum_{i=1}^{n}1(\tilde{S}(X)=1)\overset{ˆ}{\phi }\left(x_{i},a_{i},y_{i}\right)}{\sum_{i=1}^{n}1(\tilde{S}(X)=1)}$$

(1) $$\text{s.t.}\;E[\tilde{S}(X)]\geq c\;\alpha \leq e(x)\leq 1-\alpha ,\forall x:\tilde{S}(x)=1,$$

where c∈(0,1) is the desired subgroup size, and α∈[0,0.5) is the threshold controlling the overlap constraint. Beyond the size and overlap, our method naturally accommodates more general **linear and ratio-form constraints**, which we formally introduce in Lemma 2 and Remark 2 (Section 4.2).

### 3.2 Relaxing the Combinatorial Formulation

Since Problem I is combinatorial and difficult to optimize, we relax the subgroup identification to a probabilistic assignment. This relaxation is implemented using a parametric surrogate model $S:R^{d}\mapsto (0,1)$ with parameters θ. The ATE on the identified subgroup can be expressed as

(2) $$f(\theta )=\frac{\sum_{i=1}^{n}S\left(x_{i};\theta \right)\overset{ˆ}{\phi }\left(x_{i},a_{i},y_{i}\right)}{\sum_{i=1}^{n}S\left(x_{i};\theta \right)}.^{1}$$

Let $\overset{ˆ}{e}\left(x_{i}\right),{\overset{ˆ}{\mu }}_{a}\left(x_{i}\right)$ denote the estimated $e\left(x_{i}\right)$ and $\mu_{a}\left(x_{i}\right)$. We then adopt the AIPTW estimator, which can be expressed as functions of $\overset{ˆ}{e}\left(x_{i}\right)$ and ${\overset{ˆ}{\mu }}_{a}\left(x_{i}\right)$:

(3) $${\overset{ˆ}{\phi }}_{\text{aiptw}}\left(x_{i},a_{i},y_{i}\right)={\overset{ˆ}{\mu }}_{1}\left(x_{i}\right)-{\overset{ˆ}{\mu }}_{0}\left(x_{i}\right)+\frac{a_{i}}{\overset{ˆ}{e}\left(x_{i}\right)}\left(y_{i}-{\overset{ˆ}{\mu }}_{1}\left(x_{i}\right)\right)-\frac{1-a_{i}}{1-\overset{ˆ}{e}\left(x_{i}\right)}\left(y_{i}-{\overset{ˆ}{\mu }}_{0}\left(x_{i}\right)\right).$$

While this study focuses on AIPTW, $\overset{ˆ}{\phi }\left(x_{i},a_{i},y_{i}\right)$ can be derived using other ATE estimators, making this formulation flexible. More details are illustrated in Appendix C.1.

Due to the relaxation of subgroup identification into a probabilistic assignment, the overlap constraint in (1), originally designed for discrete subgroup selection, must be adapted. To achieve this, we introduce $h\left(x_{i},\alpha \right)$, a surrogate function that reformulates the overlap constraint from Problem I:

(4) $$h\left(x_{i},\alpha \right)=1-\frac{\overset{ˆ}{e}\left(x_{i}\right)\left(1-\overset{ˆ}{e}\left(x_{i}\right)\right)}{\alpha (1-\alpha )}.$$

The following result, Lemma 1 (proof in Appendix B.1), establishes that the overlap constraint in Problem I can be replaced by a constraint on $h\left(x_{i},\alpha \right)$:

**Lemma 1.** $S\left(x_{i};\theta \right)h\left(x_{i},\alpha \right)\leq 0$ *if and only if* $\alpha \leq \overset{ˆ}{e}\left(x_{i}\right)\leq 1-\alpha$.

With Lemma 1, our optimization can be reformulated as:

(Problem II) $$\underset{\theta }{\text{min}}\;-f(\theta )$$

$$\text{s.t.}\;\frac{1}{n}\sum_{i=1}^{n}S\left(x_{i};\theta \right)\geq c,\;S\left(x_{i};\theta \right)h\left(x_{i},\alpha \right)\leq 0,\forall i.$$

Solving Problem II remains a significant challenge. As shown in (2), the parametric model $S\left(x_{i};\theta \right)$ appears in both the numerator and denominator, making the objective function f(θ) neither convex nor concave, even for simple models like logistic regression. This nonconvexity also extends to the feasible set, rendering standard convex optimization methods, such as ADMM and FW, unsuitable. While Lagrangian relaxation could in principle be applied, doing so would require tuning a separate multiplier for each constraint, making the hyperparameter tuning process impractical at scale. To address this, we reformulate Problem II and present the final framework in Section 4.

## 4 Optimization Methods

Section 4.1 reformulates the task as a min-max optimization and adopts the γ-*Gradient Descent Ascent* (γ-GDA) algorithm [46]. Section 4.2 establishes feasibility guarantees, showing that MOSIC can identify the optimal subgroup while satisfying multiple constraints.

### 4.1 Min-Max Formulation and GDA

Since neither the objective nor the feasible region in Problem II is convex, we rewrite it using the saddle-point formulation:

(5) $$L\left(\theta ,\lambda \right):=-f\left(\theta \right)+\lambda^{T}g\left(\theta \right),\;\underset{\theta }{\text{min}}\;\underset{\lambda \geq 0}{\text{max}}\;L(\theta ,\lambda ).$$

We note that this is solving the primal constrained problem directly through a *min-max Lagrangian formulation*, unlike Lagrangian relaxation, which operates on the dual problem. We can solve this problem by γ-GDA [26], as described in Algorithm 1. The solution to Eq. (5) is equivalent to solving Problem II [9].

While the saddle-point formulation provides a correct representation of Problem II, applying standard GDA can lead to numerical instability and hinder convergence. Consider a scenario with a single group size constraint $\left(c-\frac{1}{n}\sum_{i=1}^{n}S\left(x_{i};\theta \right)\right)$ and its Lagrange multiplier $\lambda_{n+1}$. Initially, the constraint is violated because the group size is below the threshold, leading to a positive gradient that increments $\lambda_{n+1}$ at each iteration. Once the constraint is met, the gradient becomes negative, driving $\lambda_{n+1}$ toward 0. However, due to the small learning rate, $\lambda_{n+1}$ remains nonzero in subsequent iterations, continuing to penalize the objective and leading to artificially shrunk feasible regions. This instability extends to multiple constraints, causing the algorithm to oscillate between a strictly feasible but suboptimal θ and an optimal but infeasible θ, undermining convergence. To obtain stable and convergent γ-GDA dynamics, additional structural conditions are needed. We thus refine the objective to satisfy two key properties:

- Only violated constraints contribute gradients. This can be achieved by introducing a ReLU transform on the constraint vector. When $g_{i}(\theta )\leq 0$ (i.e., the constraint is satisfied), the penalty term becomes zero, so satisfied constraints no longer affect the optimization dynamics.
- Ensuring convergence to a feasible, locally optimal solution.

For the second requirement, we begin by defining local optimality in the context of a nonconvex-nonconcave min-max problem, introducing the concept of *local minmax point* (Definition 1). Informally, it is a fixed point where the objective remains stable under small perturbations in θ (the parameters over which we minimize) and worst-case perturbations in λ (the parameters over which we maximize). Theorem 1 [26] states that if the min-max objective function is twice differentiable, then the γ-GDA algorithm, upon convergence, reaches either a local minmax point or a stationary point with a degenerate Hessian.

However, the Hessian of our objective in (5) is inherently degenerate: $\nabla_{\lambda \lambda }^{2}L(\theta ,\lambda )=0$, which hinders guarantees of local optimum convergence and feasibility. Unlike Nandwani et al. [40], who did not exclude degenerate points–potentially invalidating their results–we introduce a modification that eliminates degenerate points, yielding the final objective of MOSIC:

(Problem III) $$\underset{\theta }{\text{min}}\;\underset{\lambda \geq 0}{\text{max}}\;L(\lambda ,\theta ),$$

$$L(\lambda ,\theta )=-f(\theta )+\lambda^{T}\text{Re}LU(g(\theta ))-\frac{\beta }{2}\lambda^{2},$$

where $\lambda \in R_{+}^{n+1},g(\theta )=\left(S\left(x_{1};\theta \right)h\left(x_{1};\alpha \right),\ldots ,S\left(x_{n};\theta \right)h\left(x_{n};\alpha \right),c-\right.\frac{1}{n}\sum_{i=1}^{n}S{\left(x_{i};\theta \right)}^{⊤}$. For notational convenience, we write g(θ) without explicitly indicating its dependence on fixed constraint values c and α (or those for any additional constraint, if present).

### 4.2 Feasibility Guarantees

With Problem III, we establish that if Algorithm 1 converges, it reaches a *strict local minmax* point (Proof in Appendix B.2):

**Proposition 1** (Local Optimality). *Let* $\left(\theta^{\prime },\lambda^{\prime }\right)$ *be a* linearly stable point *(formally defined in Definition 2) of* Algorithm 1*. Then*, $\left(\theta^{\prime },\lambda^{\prime }\right)$ *must be a* strict local minmax point.

Building on this, the following result, Lemma 2, shows that upon convergence, all constraints are approximately satisfied within a small tolerance. This guarantee applies not only to the group size constraint but also to general linear constraints in S (though not linear in θ), which include the overlap constraint.

**Algorithm 1:** **γ**-Gradient Descent Ascent (γ-GDA)

1:	**Input:** step size η, decay rate ζ, objective function L(θ,λ).
2:	Initialize $\lambda_{0}=0$; Initialize $\theta_{0}$ randomly
3:	**for** t=0,1,…T **do**
4:	If converged, output $\theta^{*}=\theta_{t}$
5:	$\gamma \leftarrow (1+t{)}^{\zeta }$
6:	Update $\theta_{t}$ using gradient descent with learning rate $\eta /\gamma :\theta_{t+1}\leftarrow \theta_{t}-\left(\frac{\eta }{\gamma }\right)\nabla_{\theta }L\left(\theta_{t},\lambda_{t}\right)$.
7:	Update $\lambda_{t}$ using gradient ascent with learning rate $\eta :\lambda_{t+1}\leftarrow \lambda_{t}+\eta \nabla_{\lambda }L\left(\theta_{t+1},\lambda_{t}\right)$.
8:	**end for**
9:	**Output:** $\theta^{T}$

**Lemma 2.** *Suppose the constraints include a group size constraint*, $g^{\text{size}}(\theta )=c-\frac{1}{n}\sum_{i=1}^{n}S\left(x_{i};\theta \right)$, *and* K(K≥0) *additional constraints linear in* $S,g^{k}(\theta )=a^{k}+\sum_{i=1}^{n}b_{i}^{k}\;S\left(x_{i};\theta \right)$, *where* $a^{k},b_{i}^{k}\in R$, *and*, $\forall k,\left|\sum_{i=1}^{n}b_{i}^{k}\right|>0$. *Together, they define the constraint vector* $g(\theta )={\left(g^{1}(\theta ),\ldots ,g^{K}(\theta ),g^{\text{size}}(\theta )\right)}^{⊤}$.

*Define* ξ>0 *as the tolerance*, $\phi_{\text{max}}={\text{max}}_{i}\;\overset{ˆ}{\phi }\left(x_{i},a_{i},y_{i}\right),L=\text{sup}\left|\partial S(\cdot ;\theta )/\partial \theta_{j}\right|$ *as the coordinate-wise Lipschitz constant, and* $\mu_{\Delta }=E\left[\partial S\left(x_{i};\theta \right)/\partial \theta_{j}\right]$ *for* $j=\text{arg}\;{\text{max}}_{j}\left|\mu_{\Delta }\right|/L$.

*Let* $\left(\theta^{*},\lambda^{*}\right)$ *be a strict local min-max point obtained by* Algorithm 1*. If*

$$\beta <\frac{\xi (c-\xi )\left|\mu_{\Delta }\right|}{2\phi_{\text{max}}L},$$

*then either the model collapses* $\left(\frac{1}{n}\sum_{i=1}^{n}S\left(x_{i};\theta^{*}\right)<\xi \right)$ *or all constraints are approximately satisfied:*

$$g^{\text{size}}\left(\theta^{*}\right)\leq \xi ,\;\text{and furthermore, with probability at least}\;1-\delta ,\;g^{k}\left(\theta^{*}\right)\leq \frac{\xi }{\left|\sum_{i=1}^{n}b_{i}^{k}\right|\left(1+\frac{L}{\left|\mu_{\Delta }\right|\sqrt{n}}\sqrt{\text{log}\;\frac{2}{\delta }}\right)},\forall k\in [K]$$

The proof of Lemma 2 (Appendix B.3) analyzes each constraint at *strict local minmax points* and establishes that, with β properly chosen,^2^ the constraints are either fully satisfied or violated up to a small tolerance error, if the model does not collapse. The proof further shows that, as long as the feasible region is non-negligible, the model is unlikely to collapse.^3^

**Remark 1** (Implication on the overlap constraint). *When* n→∞, *the bound on constraint violation is governed by* $\left|\sum_{i=1}^{n}b_{i}^{k}\right|$. *For the overlap constraint on sample* j, *this term reduces to* $h\left(x_{j};\alpha \right)$ *since* $b_{i}^{k}=1(i=j)h\left(x_{j};\alpha \right)$. *When* $h\left(x_{j};\alpha \right)$ *is small, this denominator inflates the bound, suggesting potentially high violation. However, a small* $h\left(x_{j};\alpha \right)$ *indicates that the corresponding violation of the overlap condition is itself negligible. Thus, such constraints can be safely ignored in practice*.

**Remark 2** (Extension to ratio constraints). *Because the model maximizes the subgroup ATE, it naturally favors smaller subgroups by excluding samples with relatively low estimated CATE, while the group size constraint enforces a group size lower bound c*. *As a result, the group size typically converges to the size threshold c, i.e.,* $\sum_{i=1}^{n}S\left(x_{i};\theta \right)\approx c$ *at termination. This observation allows us to extend the linear constraint to ratio constraints*

$$g^{k}(\theta )=a^{k}+\frac{\sum_{i=1}^{n}b_{i}^{k}S\left(x_{i};\theta \right)}{\sum_{i=1}^{n}S\left(x_{i};\theta \right)}.$$

*To retain compatibility with Lemma 2, we block gradient flow through the denominator during backpropagation, effectively treating it as a constant*.

**Algorithm 2:** MOSIC

1:	**Input:** ${\left\{\left(x_{i},a_{i},y_{i}\right)\right\}}_{i=1}^{n}$, constraint-related values c and α, learning rate η, decay rate ζ
2:	Estimate ${\overset{ˆ}{\mu }}_{0}(X),{\overset{ˆ}{\mu }}_{1}(X),\overset{ˆ}{e}(X)\%$ *Estimate nuisance functions*
3:	Compute $\left.{\overset{ˆ}{\phi }}_{(}x_{i},a_{i},y_{i}\right)$ using nuisance functions *% Pseudo-outcomes*
4:	Construct L(θ;λ;c,α)
5:	$\theta^{*}\leftarrow \gamma \text{-GDA}(\eta ,\zeta ,L(\theta ;\lambda ;c,\alpha ))$ *% Solve* Problem III *using* Algorithm 1
6:	**Output:** Parametric surrogate model $S\left(X;\theta^{*}\right)$

The linear and ratio families capture many practical constraints relevant to healthcare applications. Linear constraints include the overlap constraint and budget constraints (each patient incurs a treatment cost under limited resources). Ratio constraints cover safety constraints (e.g., risk levels associated with patients) and certain fairness constraints (e.g., conditional statistical parity metric). Section 5.3 assesses MOSIC’s extendability to these constraints. Due to the nonconvexity of both the objective and the feasible region, extending the guarantee to more complex constraints remains an open direction for future work.

Finally, we summarize our overall framework, MOSIC, in Algorithm 2. It first estimates the nuisances and computes the pseudo-outcomes for each sample, which are then fed into the objective function (Problem III). The optimization is subsequently performed using the γ-GDA algorithm.

## 5 Experiments

We evaluate MOSIC on both synthetic and real-world data. Section 5.1 outlines the experimental setup. Section 5.2 demonstrates that MOSIC achieves high ATE while maintaining comparable covariate balance, or vice versa. Section 5.3 further examines the robustness, interpretability, extensibility, and computational properties of MOSIC.

### 5.1 Setup

#### Datasets.

We evaluate MOSIC on synthetic and real-world datasets:

1. We generate synthetic data following a procedure adapted from Assaad et al. [2] (details in Appendix C.2). We introduce an *imbalance parameter* $\tilde{\omega }\geq 0$ to determine the strength of confounding bias. In particular, we generated two datasets of size n=5,000 with d=10 covariates and the continuous outcome Y: (1) one with no confounding bias $\left(\tilde{\omega }=0\right)$ and (2) one with high confounding bias $\left(\tilde{\omega }=5\right)$.
2. We use two de-identified datasets from intensive care units (ICU): The eICU dataset (n=13,361,d=23 covariates) [42] and the MIMIC-IV (n=6,516, same covariates) [27]. In both datasets, treated patients (A=1) received an initial Glucocorticoids dose of 160mg within 10 hours before to 24 hours after ICU admission. The outcome Y represents 7-day survival, with Y=1 indicating survival and Y=0 otherwise. The covariates X include lab test results, vital signs, and sequential organ failure assessment (SOFA) scores [54].

#### Baselines.

We compare MOSIC with two categories from Section 2: those designed for subgroup identification and those adapted from CATE estimation algorithms.

1. Dedicated subgroup identification methods: We evaluate CAPITAL, OWL, and VT, modifying them to incorporate a group size constraint via thresholding (Details in Appendix C.3). These methods prioritize interpretability, making it unclear whether performance limits stem from this trade-off or poor CATE estimation. To address this, we consider the next category.
2. CATE estimation algorithms: We evaluate three methods: CT, CF, and Dragonnet in the main results. We additionally compare with DR-learner, R-learner, BART, and an overlap-weighted variant of MOSIC (MOSIC-OW) in Appendix E.4. In these baselines, patients are ranked by the estimated CATE values, and the top subgroup of the desired size is selected [53]. While not designed for subgroup selection, they provide a natural baseline. If CATE estimation were accurate, this approach would identify the optimal subgroup, allowing us to separate the impact of CATE estimation reliability from the subgroup interpretability trade-off.

#### Evaluation Metrics.

We assess performance using two metrics: **(1) Subgroup ATE**, which measures whether identified subgroups achieve high ATE at the desired size. On synthetic data, we compute the ground-truth ATE; on real-world data, we use the difference between the subgroup and overall AIPTW estimates. **(2) ATE Estimation Reliability.** On synthetic data, it is measured by the AIPTW estimation error. On real-world data, where the true ATE is unobserved, it is measured by the number of unbalanced features. A feature is considered unbalanced [15] if its standardized mean difference (SMD) > 0.2 after IPTW reweighting [5, 58]. More unbalanced features indicate greater estimation uncertainty and lower subgroup reliability. To validate imbalance as a proxy for estimation error, we also report the correlation of estimation error and feature imbalance on synthetic data (Appendix E.1). To assess how well MOSIC enforces the overlap constraint, we further measure the proportion of test-set samples that violate the overlap constraint on the real-world data.

#### Implementation Details.

For all datasets, we perform 100 random splits of the training and test sets. For each split, we first conduct a 5-fold cross-validation on the training set to determine the optimal hyperparameters (Appendix C.5). The model is then retrained on the entire training set using the selected hyperparameters and evaluated on the corresponding test set. The final results are reported using the mean and standard deviations across all 100 evaluations.

We implement the subgroup identification model (S) using MLP and DT. In the next sections, ‘MOSIC’ refers to MOSIC-MLP, unless stated otherwise. For DTs, we adopt the neural-network representation of [38]. All these models are trained using Algorithm 1, with L1 regularization applied in the loss function. For nuisance function estimation, we use LR for the propensity score model ê(·), and Dragonnet for the outcome models ${\overset{ˆ}{\mu }}_{a}(\cdot )$ (Appendix C.4). ^5^ Our implementation shares the training data for nuisance estimation and subgroup selection. But the final comparison on the test set is not affected by such sharing because the test set is never used for either step.

### 5.2 Results

#### Synthetic Data.

To assess the impact of overlap constraints, we compare MOSIC with (α=0.02) and without (α=0) them. Figure 1 demonstrates that MOSIC consistently identifies subgroups with the highest true subgroup ATE across all group sizes (Figure 1(a) and 1(b), left); and it achieves the lowest ATE estimation errors (Figure 1(a) and 1(b), right). We also investigate the statistical properties of MOSIC in Appendix F and G.

#### Real-World Data.

Since the real-world datasets contain a large portion of samples with propensities outside [0.05, 0.95] (Figure E.5), we relax the overlap constraint threshold to be α=0.01. Figures 2 and Figure E.4 demonstrate that MOSIC consistently outperforms other methods, and Table E.1 quantifies the statistical significance of the performance differences shown in Figure 2. We highlight the importance of jointly evaluating subgroup ATE and covariate balance when assessing performance. A large ATE alone is insufficient if covariate imbalance persists, as it may indicate unreliable estimates. As shown in Figures 2(a) and 2(b) (left), MOSIC achieves higher subgroup ATEs at comparable group sizes in most cases. Even when the ATE advantage is not statistically significant (e.g., MOSIC (α=0.01) vs. CF at c=0.6 on MIMIC, p = 0.68), MOSIC delivers significantly better covariate balance (p = 5.1E-04; Figures 2(a) and 2(b), right).

To quantify the uncertainty of the ATE, we additionally compute 95% confidence intervals for the subgroup ATEs and conduct a sensitivity analysis of unmeasured confounding (Appendix E.3). For completeness, feature imbalance with SMD> 0.1 as the threshold is also presented in Appendix E.7.

Figure 3 (right) shows that MOSIC with the overlap constraint successfully limits the number of test-set samples falling outside the allowable propensity range, demonstrating effective enforcement of the constraint (additional results are provided in Appendix E.6). This reduction in extreme-propensity samples leads to improved feature balance on eICU (Figures 2(a), right).

#### Alternative Outcome Definition.

To evaluate robustness to the outcome specification, we additionally consider ventilation-free days within 7 days of ICU admission, assigning 0 ventilation-free days to patients who die within 7 days. Table 1 shows that the subgroup ATEs identified by MOSIC and the baselines are largely comparable. However, MOSIC consistently achieves substantially better covariate balance, reducing the number of unbalanced features by 25–40% across subgroup sizes. Additional balance diagnostics in Appendix E.8 confirm this trend. These results are consistent with our main findings and suggest that the benefit of MOSIC is robust to the choice of outcome definition.

### 5.3 Additional Analyses

#### Incorporating Sample-Splitting.

To assess whether internal samplesplitting improves subgroup learning, we implemented 5-fold crossfitting on the training data: nuisances were trained on 4 folds and used to generate CATEs on the held-out fold, and the subgroup model was trained on these cross-fitted CATEs. Evaluation remained on the untouched test set, where nuisances were refit on the full training set before computing AIPTW. The results (Figure 4) are similar to our original pipeline, suggesting that the benefit of decoupling nuisance and subgroup estimation is limited in our setting, likely because further splitting of a modest dataset weakens nuisance estimation.

#### Decision Tree as Backbone.

While MOSIC-MLP outperforms baselines, its black-box nature limits interpretability. In contrast, decision trees are favored in clinical settings for their transparency [10]. We therefore compare MOSIC with DT backbone to DT-based baselines (CT, CF) on eICU. VT is excluded due to its high ATE estimation error on synthetic data (Figure 1(b)).

To impose a fair comparison, we match model capacity: we fix the tree depth to 5 for both MOSIC (denoted as MOSIC-DT) and CT, and use ensembles of 3 trees of depth 5 for MOSIC (denoted as MOSIC-Forest) and CF. As shown in Figure 5, MOSIC consistently outperforms CT and CF under the same interpretability requirement. Notably, despite the limited model capacity, MOSIC effectively enforces both group size and overlap constraints, leading to improved covariate balance.

#### Extension to Additional Constraints.

In addition to the group size and overlap constraint introduced earlier, MOSIC readily extends to other constraints. We demonstrate this capability on both synthetic and real-world data. On synthetic data, we progressively impose further requirements on top of the size and overlap constraints: first adding a safety constraint, then safety and budget constraints, and finally safety, budget, and fairness constraints. Table 2 demonstrates that MOSIC can effectively satisfy all of them (Detailed specification of these constraints is provided in Appendix E.9).

On eICU, in addition to the size (c=0.4) and overlap constraint (α=0.01), we introduced a safety constraint motivated by evidence that glucocorticoids may exacerbate neural damage [24]. In particular, we required that the proportion of patients with a Glasgow Coma Scale (GCS) score < 6 remain below 0.05. GCS measures the level of consciousness, with lower scores indicating more severe dysfunction. We targeted patients with GCS < 6 because this threshold represents the most severe central nervous system dysfunction in the SOFA, the most commonly used ICU metric [49]. Without a constraint on the GCS score, we observed that the selected subgroup contained a nontrivial number of patients with GCS< 6. Since observational studies have suggested that glucocorticoids may exacerbate neural system damage [24], such a subgroup raises a safety concern. We therefore limit, rather than strictly exclude, such patients, acknowledging that a small subset with severe neurological impairment may still derive benefit from treatment.

We evaluate both MOSIC-DT and MOSIC-MLP with this additional constraint (See Appendix E.10 for details). Table 3 shows that MOSIC-DT can additionally satisfy this safety constraint even under the interpretability restriction. The resulting DT examples show that MOSIC-DT indeed introduces an explicit rule to exclude low-GCS patients (Figure 6(b)), in contrast to the result before adding this constraint (Figure 6(a)). Since DTs may be sensitive to randomness, we additionally run MOSIC-MLP with and without the GCS< 6 constraints and run post-hoc SHAP-value analysis to evaluate the feature contributions. Figure 6 confirms that the dominant features identified by SHAP are generally consistent with the rule structure revealed by the decision tree. In particular, the SHAP values of the GCS features were ambiguous before we enforced the constraint that avoids patients with GCS < 6 (Figure 6(c)), but they became clearly separated after we enforced it (Figure 6(d)), aligning well with our intention.

#### Discussion on an identified subgroup rule.

To provide insight into the characteristics of the identified subgroup, we examine one learned subgroup rule. Fig. 6(a) shows that MOSIC-DT primarily selects patients with higher respiratory severity and lower temperature. The respiratory component is clinically plausible, as corticosteroids are known to benefit patients with severe pulmonary inflammation, such as ARDS and pneumonia [12, 51]. The SHAP analysis of MOSIC-MLP (Fig. 6(c)) reveals a similar pattern, assigning higher importance to chronic lung disease, elevated respiratory rate, and indicators of respiratory severity. In contrast, the temperature signal is less straightforward. While hypothermia in sepsis is associated with worse outcomes and dysregulated host response [7], it is not an established marker of corticosteroid benefit. Overall, some subgroup characteristics align with existing clinical knowledge, whereas others warrant further investigation.

#### Runtime Analysis and Training Dynamics.

Gradient-based methods such as GDA scale well in high-dimensional settings, with per-iteration cost linear in the number of parameters. This makes MOSIC more scalable than combinatorial approaches such as CAPITAL. Although MOSIC involves many constraints, its overhead is modest: each constraint requires a single evaluation per update, resulting in a cost of O(nm) for m constraints and n samples. Moreover, the n overlap constraints contribute only O(n) operations in total, since each involves a single multiplication. Consequently, MOSIC maintains a low per-iteration cost despite the large number of constraints. Figure 7 further confirms stable optimization under multiple constraints.

We also provide an empirical runtime comparison (Appendix H). To reflect resource-limited clinical environments, all experiments are conducted on CPUs. Table 4 shows that the optimization overhead is small relative to nuisance estimation, suggesting that computation is unlikely to be a practical deployment bottleneck.

## 6 Conclusion

We propose MOSIC, a model-agnostic framework for optimal subgroup identification that handles multiple constraints with feasibility guarantees. It demonstrates strong empirical performance under group size and overlap constraints, flexibly extends to additional clinical constraints, and supports diverse models to deliver interpretable solutions.

Finally, we acknowledge that the real-world ICU datasets used in this study have known limitations, including potential immortal-time bias and unmeasured confounding. Therefore, subgroup rules obtained from these datasets (e.g., Fig. 6) should be interpreted as hypothesis-generating rather than clinically prescriptive. These intrinsic limitations affect all subgroup identification and CATE-based approaches equally, and do not affect the conclusion of our comparison. In addition, our ablation study using a GCS-based safety constraint demonstrates how domain knowledge can be integrated to refine subgroup definitions when needed.

## 7 GenAI Disclosure

We used generative AI tools solely for language polishing (e.g., grammar and clarity edits). All scientific content, including problem formulation, methodology, theory, experiments, results, and conclusions, was developed and verified by the authors.

## 8 Limitations and Ethical Considerations

This study uses publicly available, de-identified ICU datasets (eICU and MIMIC-IV) released for research under established data use agreements; no re-identification was attempted. The analyses do not involve direct interaction with human subjects and are conducted in accordance with the datasets’ policy. Methodologically, results are based on observational data and standard causal assumptions (e.g., unconfoundedness and overlap), so identified subgroups and estimated effects should be interpreted as exploratory rather than clinical guidance. The proposed method is intended for research and decision support, not for direct clinical deployment without further validation and oversight.

## Supplementary Material

1

## Figures and Tables

**Figure 1:**
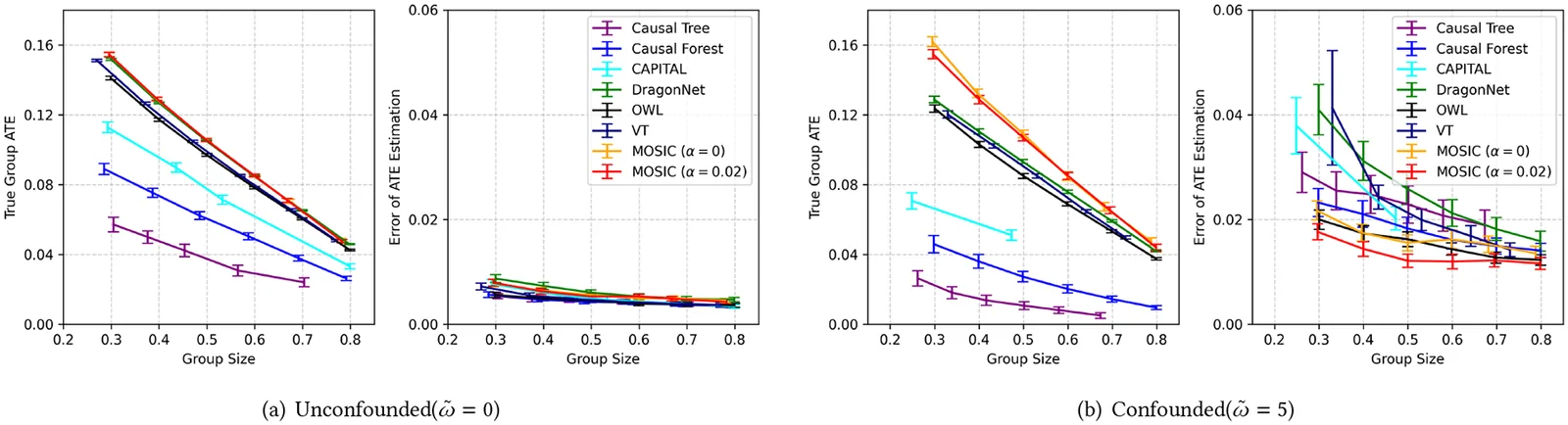
True ATE and estimation error across different group sizes on synthetic data

**Figure 2:**
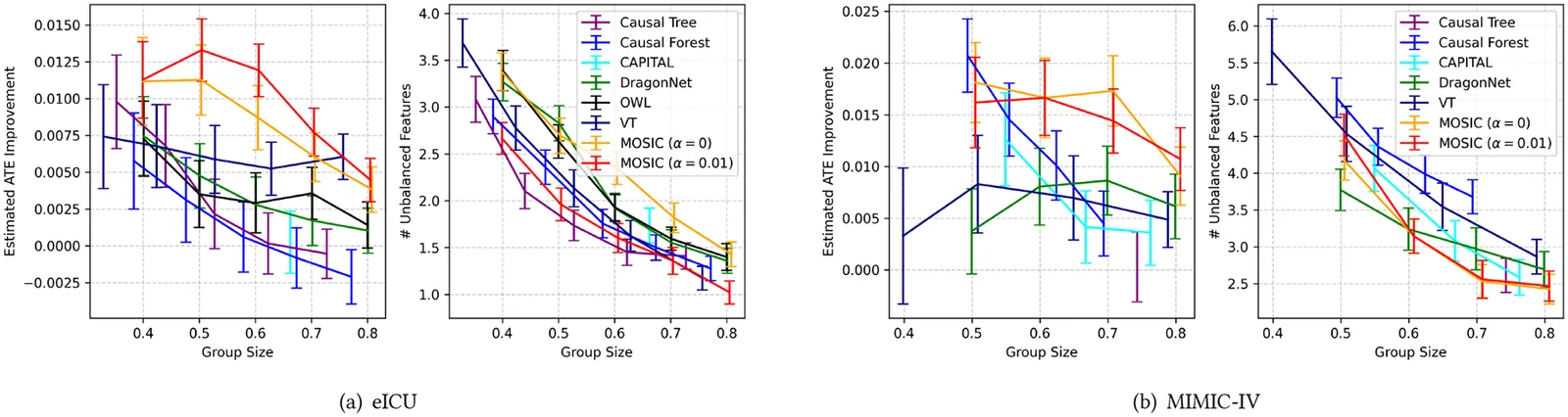
Estimated ATE and the number of unbalanced features on real-world datasets.^4^

**Figure 3:**
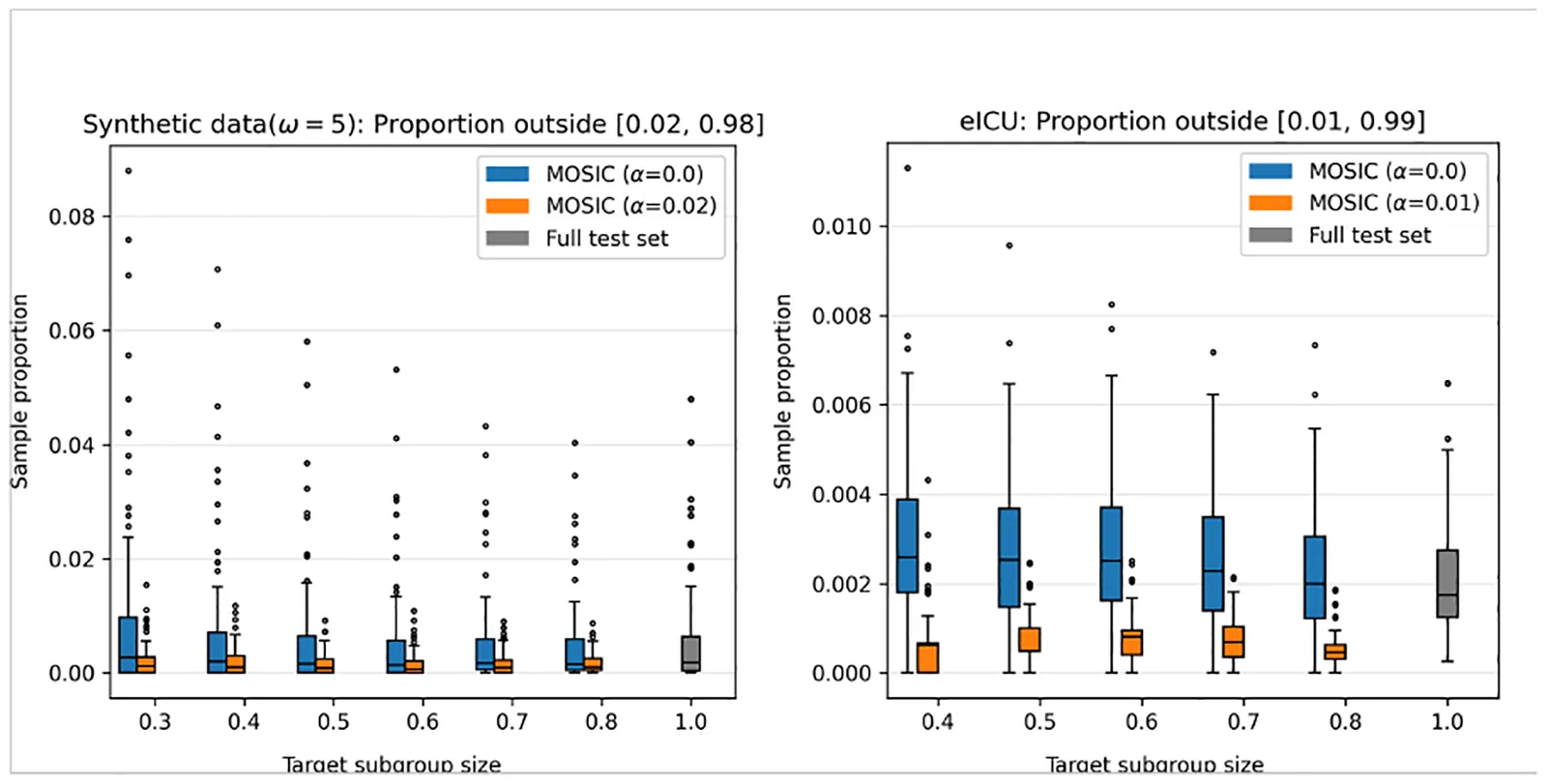
Test set sample proportion that violates the corresponding overlap constraints on confounded synthetic data (left) and eICU (right)

**Figure 4:**
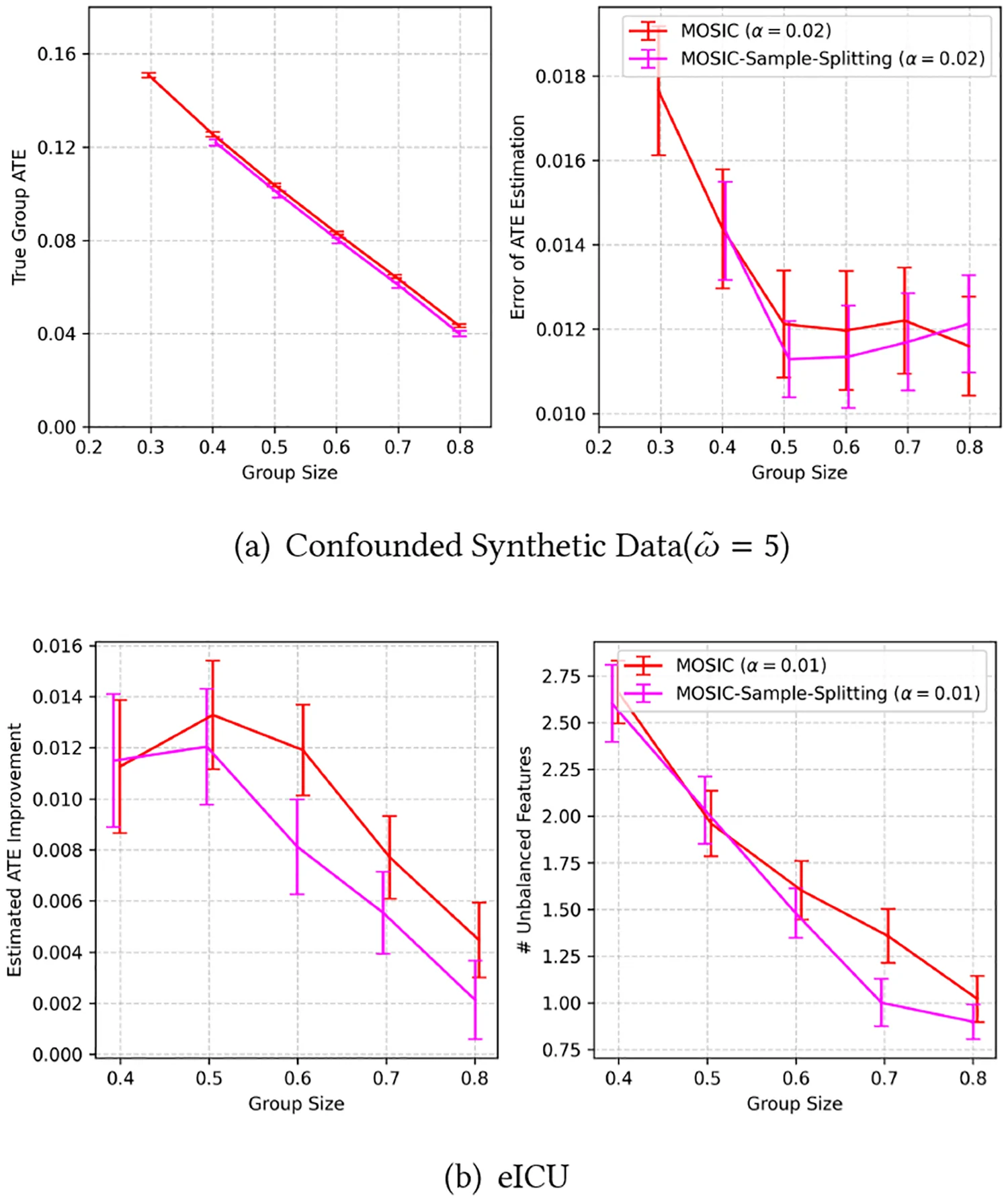
MOSIC w/ and w/o cross-fitted CATE estimates.

**Figure 5:**
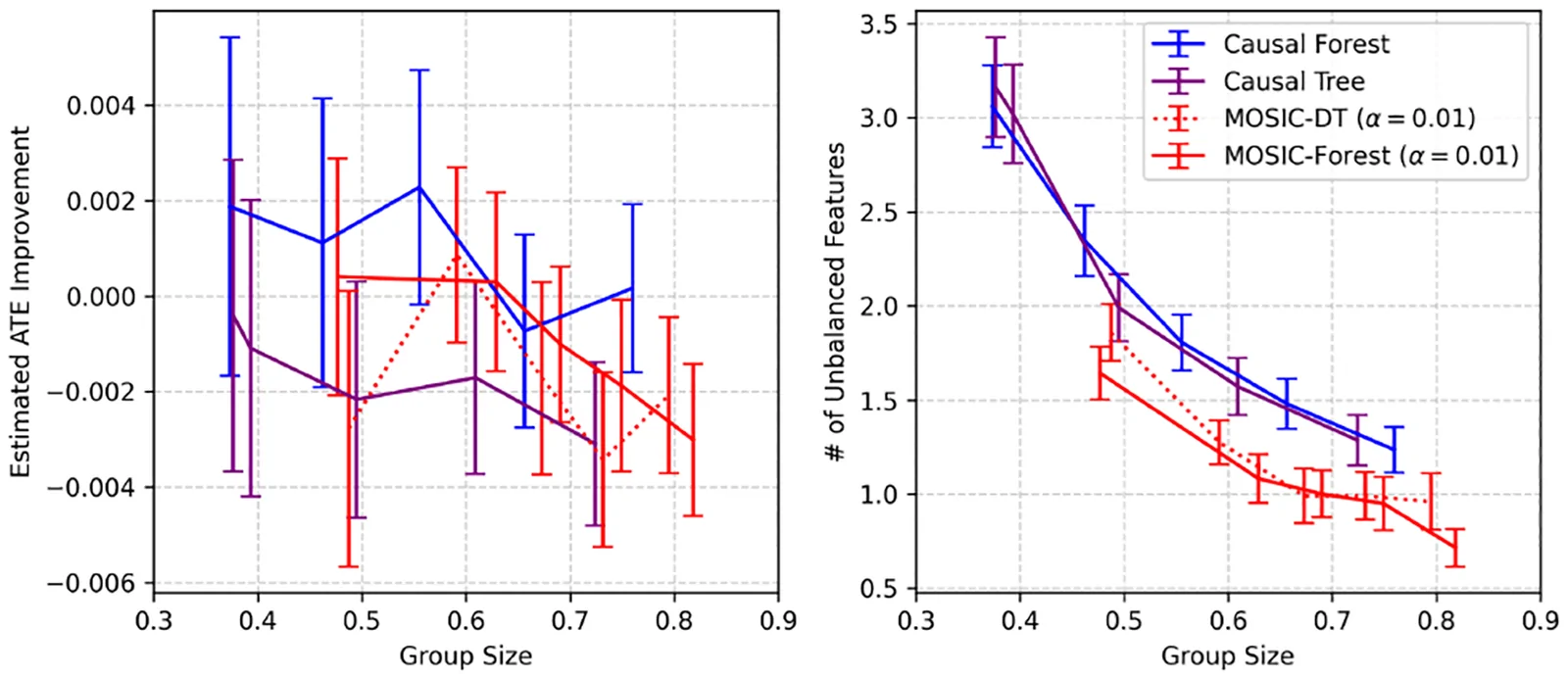
Results on eICU: MOSIC with DT backbone vs. DT-based baselines.

**Figure 6:**
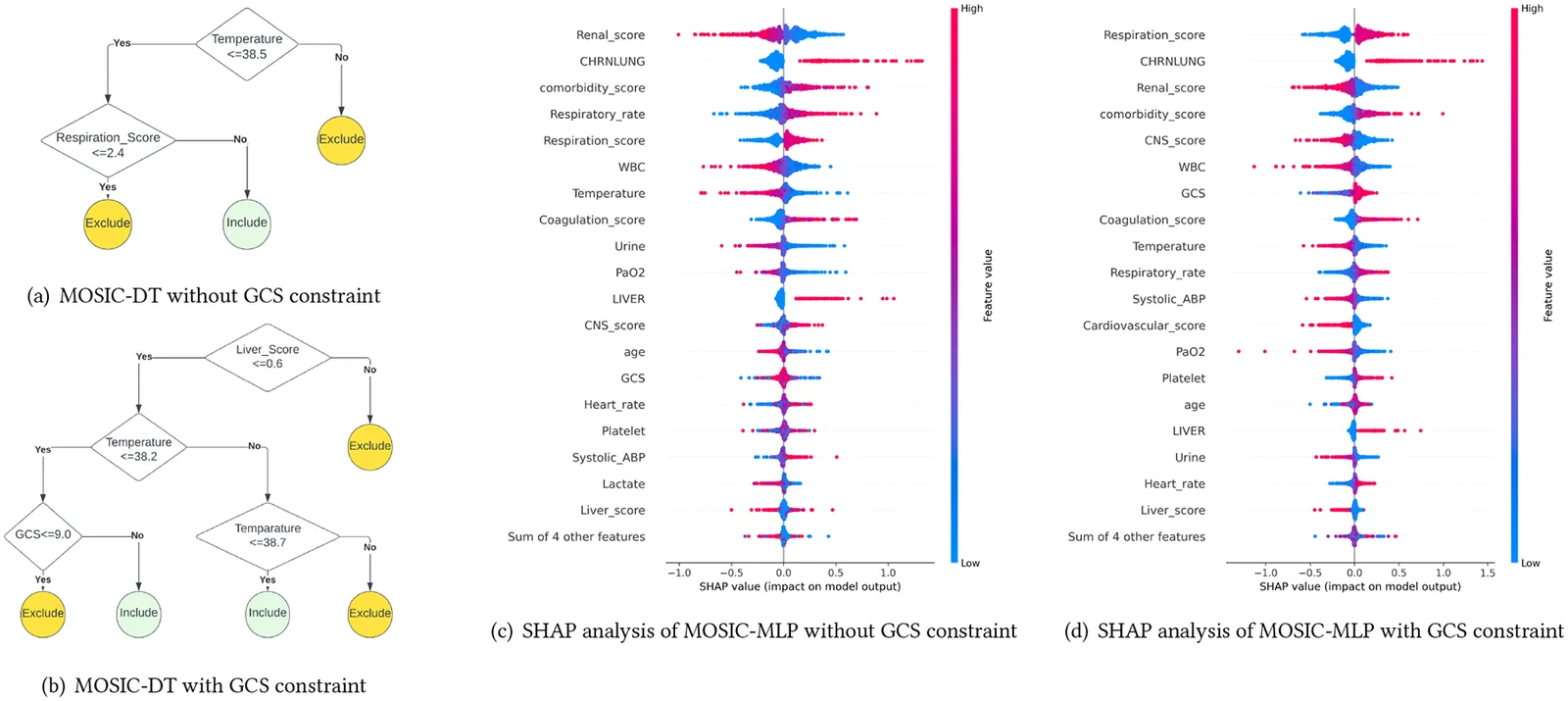
Resulting DT example of MOSIC-DT (left) and SHAP analyses of MOSIC-MLP (middle/right) on eICU with and without the additional GCS< 6 constraint. The two DTs are trained from the same split for comparison.

**Figure 7:**
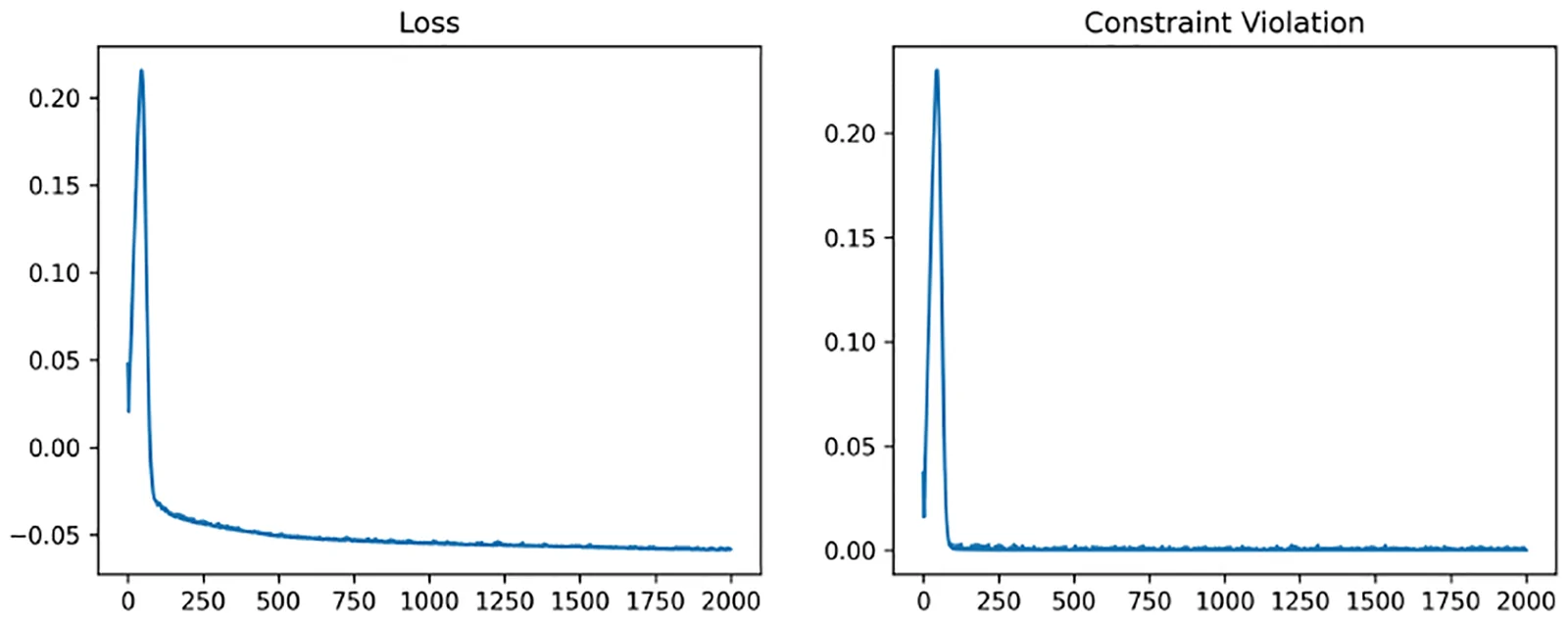
Training dynamics on eICU under size and overlap constraints. Left: training loss. Right: total constraint violation (positive values indicate violations; 0 indicates all constraints are satisfied)

**Table 1:** Alternative outcome definition: ventilation-free days within 7 days.

	Subgroup ATE			# Unbalanced Features		
Method	c=0.6	c=0.7	c=0.8	c=0.6	c=0.7	c=0.8
OWL	0.05±0.01	0.04±0.00	0.03±0.00	2.59±0.18	2.28±0.17	1.73±0.14
VT	0.04±0.01	0.04±0.01	0.04±0.01	2.67±0.19	2.20±0.17	1.88±0.16
CT	0.04±0.00	0.04±0.00	0.04±0.00	3.06±0.23	2.23±0.19	1.74±0.15
CF	0.04±0.00	0.03±0.00	0.04±0.00	2.65±0.18	2.09±0.18	1.85±0.17
Dragonnet	0.05±0.00	0.05±0.00	0.04±0.00	2.65±0.18	2.14±0.15	1.89±0.17
MOSIC-MLP	0.05±0.00	0.05±0.00	0.04±0.00	1.88±0.16	1.48±0.12	1.55±0.13
MOSIC-DT	0.04±0.00	0.04±0.00	0.04±0.00	1.52±0.13	1.50±0.13	1.43±0.13
MOSIC-Forest	0.04±0.00	0.04±0.00	0.04±0.00	1.64±0.14	1.48±0.14	1.27±0.14

**Table 2:** Performance on synthetic data $\left(\tilde{\omega }=5\right)$ under multiple additional constraints. Constraints: 1) Safety: Average Risk ≤0.05; 2) Budget: Total Cost ≤ 1250; 3) Fairness: ∣Sensitive Group Ratio - 0.5∣ ≤ 0.01.

Metric	Group Size & Overlap	Group Size & Overlap & Safety	Group Size & Overlap & Safety & Budget	Group Size & Overlap & Safety & Budget & Fairness
Group Size	0.50 ± 0.01	0.50 ± 0.05	0.48 ± 0.05	0.49 ± 0.02
# Unbalanced Features	0.14 ± 0.40	0.20 ± 0.45	0.23 ± 0.57	0.23 ± 0.45
True CATE	0.10 ± 0.01	0.10 ± 0.01	0.09 ± 0.01	0.09 ± 0.01
ATE Error	0.01 ± 0.01	0.01 ± 0.01	0.01 ± 0.01	0.01 ± 0.01
Average Risk	0.08 ± 0.01	**0.05 ± 0.01**	**0.05 ± 0.01**	**0.05 ± 0.01**
Total Cost	1377.41 ± 41.57	1365.72 ± 144.95	**1252.78 ± 132.59**	**1268.40 ± 42.44**
Sensitive Group Ratio	0.45 ± 0.03	0.45 ± 0.03	0.46 ± 0.03	0.49 ± 0.02

**Table 3:** Results on eICU with the additional constraint requiring the proportion of patients with GCS< 6 to remain below 0.05. We report the mean ± standard error over 100 random splits.

	Constraint	
Metric	Size & Overlap	Size & Overlap & GCS
Group Size	0.46 ± 0.10	0.44 ± 0.10
# Unbalanced Features	2.0 ± 1.72	2.2 ± 2.28
ATE Improvement	0.02 ± 0.02	0.02 ± 0.02
Proportion of GCS < 6	**0.15 ± 0.05**	**0.03 ± 0.03**

**Table 4:** Runtime (seconds) of each method across different sample sizes. MOSIC shares the same first-stage nuisance fitting as Dragonnet; reported times reflect MOSIC’s second-stage optimization only.

		Sample Size			
Method	Constraint	1000	3000	10000	30000
Dragonnet	-	15.77	25.86	58.22	151.32
CT	-	0.00	0.01	0.03	0.06
CF	-	0.02	0.05	0.16	0.41
VT	-	0.07	0.16	0.37	0.67
OWL	-	0.20	0.20	0.73	1.83
CAPITAL	Size	1.37	7.29	25.33	89.57
MOSIC	Size + Overlap	1.64	1.97	1.67	2.24
MOSIC	Size + Overlap + Safety	1.50	1.78	1.90	2.53
MOSIC	Size + Overlap + Safety + Budget	2.34	1.78	1.74	2.43
MOSIC	Size + Overlap + Safety + Budget + Fairness	2.35	2.04	1.64	2.44

## CCS Concepts

• **Applied computing → Multi-criterion optimization and decision-making; Health informatics.**

## Footnotes

2 Constraint violation is insensitive to $\beta ;\beta \in \left[10^{-5},10^{-2}\right]$ works well.

3 If the model collapses to a near-zero subgroup, we restart with a new seed; persistent collapse indicates the constraint-defined feasible region should be re-evaluated.

4 The OWL method failed to converge on the MIMIC-IV dataset and was therefore omitted from the figure.

5 Empirically, estimating propensity scores with Dragonnet results in a large number of unbalanced features; we therefore adopt LR, demonstrating the flexibility of MOSIC.
