# Supplementary material for: MOSIC: Model-Agnostic Optimal Subgroup Identification with Multi-Constraint for Improved Reliability
Source: KDD. Author manuscript; Available in PMC 2026 Oct 13. (PMC13655699; doi:10.1145/3770855.3818926)
Supplement: Supplementary file 1 [file NIHMS2214664-supplement-1.pdf]

A Preliminary Definitions and Theorems

A.1 Local minmax point

Definition 1 (Local minmax point). *A point $(\theta^\star, \lambda^\star)$ is said to be a local minmax point of L, if there exists $\delta_0 > 0$ and a continuous function h satisfying $h(\delta) \to 0$ as $\delta \to 0$, such that for any $\delta \le \delta_0$, and any (θ, λ) satisfying*

$$\|\theta - \theta^\star\| \le \delta \quad \text{and} \quad \|\lambda - \lambda^\star\| \le h(\delta),$$

we have

$$L(\theta^\star, \lambda) \le L(\theta^\star, \lambda^\star) \le \max_{\lambda' : \|\lambda' - \lambda^\star\| \le h(\delta)} L(\theta, \lambda').$$

If a point $(\theta^\star, \lambda^\star)$ satisfies

$$[\nabla^2_{\theta\theta} L - \nabla^2_{\theta\lambda} L (\nabla^2_{\lambda\lambda} L)^{-1} \nabla^2_{\lambda\theta} L] \succ 0,$$

we call it a strict local minmax point [26].

A.2 Linearly Stable Point

Definition 2 (Linearly Stable Point). *For a differentiable dynamical system $\mathbf{w}$, a fixed point $\mathbf{z}^\star$ is a linearly stable point of $\mathbf{w}$ if its Jacobian matrix $\mathbf{J}(\mathbf{z}^\star) := \left(\frac{\partial \mathbf{w}}{\partial \mathbf{z}}\right)(\mathbf{z}^\star)$ has spectral radius $\rho(\mathbf{J}(\mathbf{z}^\star)) \le 1$.*

A.3 Convergence of the γ-GDA algorithm

Theorem 1. *(Jin et al [26], Theorem 26): Given an objective: $\min_{x \, in \mathcal{X}} \max_{y \in \mathcal{Y}} f(x, y)$. For any twice-differentiable f, the strict linearly stable limit points of the γ-GDA flow are {strict local minmax points} $\cup$ {(θ,λ) | (θ,λ) is stationary and $\nabla^2_{\lambda\lambda} f(\theta, \lambda)$ is degenerate} as $\gamma \to \infty$.*

B Proofs

B.1 Proof of Lemma 1

Lemma 1. *$S(\boldsymbol{x}_i; \theta) h(\boldsymbol{x}_i, \alpha) \le 0$ if and only if $\alpha \le \hat{e}(\boldsymbol{x}_i) \le 1 - \alpha$.*

Proof. We proceed by proving both directions separately.

Forward Direction: Suppose $S(x_i; \theta) h(x_i) \le 0$. We aim to show that this implies $\alpha \le e(x_i) \le 1 - \alpha$.

$$\begin{aligned}
& S(x_i; \theta) h(x_i) \le 0 \\
\Longrightarrow\ & h(x_i) \le 0 \qquad (\text{Since } S(x_i) > 0) \\
\Longrightarrow\ & 1 - \frac{e(x_i)(1 - e(x_i))}{\alpha(1 - \alpha)} \le 0 \qquad (\text{Substituting } h(x_i)) \\
\Longrightarrow\ & \alpha(1 - \alpha) - e(x_i)(1 - e(x_i)) \le 0 \\
\Longrightarrow\ & e(x_i) - \alpha - \left(e(x_i)^2 - \alpha^2\right) \ge 0 \\
\Longrightarrow\ & (e(x_i) - \alpha)\left(1 - (e(x_i) + \alpha)\right) \ge 0 \\
\Longrightarrow\ & (e(x_i) - \alpha)\left(1 - \alpha - e(x_i)\right) \ge 0 \\
\Longrightarrow\ & \alpha \le e(x_i) \le 1 - \alpha.
\end{aligned}$$

Backward Direction: Suppose $\alpha \le e(x_i) \le 1 - \alpha$. We need to show that this implies $S(x_i; \theta) h(x_i) \le 0$.

Define the auxiliary function $q(z) = z(1 - z)$, whose derivative is given by:

$$q'(z) = 1 - 2z.$$

Thus, $q(z)$ attains its maximum at $z = 0.5$.

When $0 < \alpha \le e(x_i) \le 0.5$, we have $q'(e(x_i)) = 1 - 2e(x_i) \ge 0$, which implies that $q(e(x_i))$ is non-decreasing on $[0, 0.5]$. Consequently, from the assumption $\alpha \le e(x_i)$, we obtain:

$$q(e(x_i)) \ge q(\alpha) \implies e(x_i)(1 - e(x_i)) \ge \alpha(1 - \alpha).$$

- Similarly, for $0.5 < e(x_i) \le 1 - \alpha < 1$, we have $e(x_i)(1 - e(x_i)) \ge \alpha(1 - \alpha)$.

By combining both cases, we conclude that:

$$e(x_i)(1 - e(x_i)) \ge \alpha(1 - \alpha).$$

Dividing both sides by $\alpha(1 - \alpha)$ yields:

$$1 - \frac{e(x_i)(1 - e(x_i))}{\alpha(1 - \alpha)} = h(x_i) \le 0.$$

Since $S(x_i) > 0$, it follows that:

$$S(x_i) h(x_i) \le 0.$$

This completes the proof. □

B.2 Proof of Proposition 1

Proposition 1 (Local Optimality). *Let $(\boldsymbol{\theta}', \boldsymbol{\lambda}')$ be a* linearly stable point *(formally defined in Definition 2) of Algorithm 1. Then, $(\boldsymbol{\theta}', \boldsymbol{\lambda}')$ must be a* strict local minmax point.

Proof. The derivation of Theorem 1 relies on the Jacobian matrix and requires twice differentiability. However, our objective function incorporates ReLU activations, introducing non-differentiability at the origin. This prevents us from directly applying Theorem 1 in its standard form. Nonetheless, since the probability of encountering exact zero inputs to ReLU is negligible, our objective function remains effectively differentiable in practice when using gradient-based optimization.

Thus, the key arguments of Theorem 1 extend to our objective (**Problem III**), implying that its stable limit points must either be local minmax points or points where the second derivative is degenerate.

Further, we compute the first and second derivatives of L with respect to λ:

$$\frac{\partial L}{\partial \lambda_i} = \mathrm{ReLU}(g_i(\theta)) - \beta \lambda_i, \tag{6}$$

$$\frac{\partial^2 L}{\partial \lambda_i \lambda_j} = \begin{cases} 0 & \text{if } i \neq j, \\ -\beta & \text{if } i = j. \end{cases} \tag{7}$$

Since $\beta > 0$, the Hessian matrix $\frac{\partial^2 L}{\partial \lambda_i \lambda_j}$ is never degenerate. Applying Theorem 1, we conclude that the strict linearly stable limit points of the γ-GDA flow are precisely the set of local minmax points. □

B.3 Proof of Lemma 2

Lemma 2. *Suppose the constraints include a group size constraint, $g^{size}(\boldsymbol{\theta}) = c - \frac{1}{n}\sum_{i=1}^n S(x_i; \boldsymbol{\theta})$, and K ($K \ge 0$) additional constraints linear in S, $g^k(\boldsymbol{\theta}) = a^k + \sum_{i=1}^n b_i^k S(x_i; \boldsymbol{\theta})$, where $a^k, b_i^k \in \mathbb{R}$, and, $\forall k, |\sum_{i=1}^n b_i^k| > 0$. Together, they define the constraint vector $\boldsymbol{g}(\boldsymbol{\theta}) = (g^1(\boldsymbol{\theta}), \ldots, g^K(\boldsymbol{\theta}), g^{size}(\boldsymbol{\theta}))^\top$.*

Define $\xi > 0$ as the tolerance, $\phi_{\max} = \max_i \hat{\phi}(x_i, a_i, y_i)$, $L = \sup |\partial S(\cdot; \boldsymbol{\theta})/\partial \theta_j|$ as the coordinate-wise Lipschitz constant, and $\mu_\Delta = \mathbb{E}[\partial S(x_i; \boldsymbol{\theta})/\partial \theta_j]$ for $j = \arg\max_j |\mu_\Delta|/L$.

Let $(\boldsymbol{\theta}^*, \boldsymbol{\lambda}^*)$ be a strict local min-max point obtained by Algorithm 1. If

$$\beta < \frac{\xi(c-\xi)|\mu_\Delta|}{2\,\phi_{\max} L},$$

then either the model collapses ($\frac{1}{n}\sum_{i=1}^n S(x_i;\boldsymbol{\theta}^*) < \xi$) or all constraints are approximately satisfied:

$$g^{size}(\boldsymbol{\theta}^*) \le \xi, \text{ and furthermore, with probability at least } 1-\delta,$$
$$g^k(\boldsymbol{\theta}^*) \le \frac{\xi}{|\sum_{i=1}^n b_i^k|(1+\frac{L}{|\mu_\Delta|\sqrt{n}}\sqrt{\log\frac{2}{\delta}})}, \forall k \in [K]$$

Proof. At convergence, the following condition holds:

$$\frac{\partial L}{\partial \boldsymbol{\lambda}}\Big|_{\lambda=\lambda^*} = ReLU(\boldsymbol{g}(\boldsymbol{\theta}^*)) - \beta\boldsymbol{\lambda}^* = \mathbf{0}, \tag{8}$$

$$\frac{\partial L}{\partial \boldsymbol{\theta}}\Big|_{\theta=\theta^*} = -\frac{\partial f}{\partial \boldsymbol{\theta}}\Big|_{\theta=\theta^*} + \boldsymbol{\lambda}^* \frac{\partial ReLU}{\partial \boldsymbol{g}} \cdot \frac{\partial \boldsymbol{g}}{\partial \boldsymbol{\theta}}\Big|_{\theta=\theta^*} = \mathbf{0} \tag{9}$$

Since Eq. (8) applies component-wise to $\boldsymbol{g}(\boldsymbol{\theta}^*)$, we analyze each individual component $g(\boldsymbol{\theta}^*)$ separately. Due to ReLU, we distinguish between two cases: $g(\boldsymbol{\theta}^*) \le 0$ and $g(\boldsymbol{\theta}^*) > 0$. When $g(\boldsymbol{\theta}^*) \le 0$, Lemma 2 holds trivially. Therefore, we focus on the remaining case where $g(\boldsymbol{\theta}^*) > 0$, which means the constraint is violated and Eq. (8) and Eq.(9) simplifies to

$$g(\boldsymbol{\theta}^*) = \beta\lambda^*, \tag{10}$$

$$-\frac{\partial f}{\partial \boldsymbol{\theta}}\Big|_{\theta=\theta^*} + \lambda^* \frac{\partial g}{\partial \boldsymbol{\theta}}\Big|_{\theta=\theta^*} = \mathbf{0} \tag{11}$$

Substituting Eq.(10) into Eq.(11), we obtain

$$-\frac{\partial f}{\partial \boldsymbol{\theta}}|_{\theta=\theta^*} + \frac{g(\boldsymbol{\theta}^*)}{\beta}\frac{\partial g}{\partial \boldsymbol{\theta}}|_{\theta=\theta^*} = 0. \tag{12}$$

We note that $g(\boldsymbol{\theta}^*)$ is a scalar, meaning that each element of $\frac{\partial f}{\partial \boldsymbol{\theta}}|_{\theta=\theta^*}$ is proportional to the corresponding elements in $\frac{\partial g}{\partial \boldsymbol{\theta}}|_{\theta=\theta^*}$ by the same constant $\frac{g(\boldsymbol{\theta}^*)}{\beta} > 0$. This allows us to pick any element of $\boldsymbol{\theta}$ to analyze $g(\boldsymbol{\theta}^*)$. (Note that when $\theta \in \mathbb{R}^d$ is high-dimensional, Eq.(12) is challenging to achieve, because we need one scalar to satisfy all d equations. In practice, we observe small oscillations when the algorithm converges, which implies the algorithm finds it hard to exactly satisfy all equations.)

Denote $\phi_i = \phi(\boldsymbol{x}_i, a_i, y_i)$, $\Delta_i = \frac{\partial S(\boldsymbol{x}_i;\theta)}{\partial \theta_j}\Big|_{\theta=\theta^*} \le L$, $\mu_\Delta = \mathbb{E}\Delta_i$, where $j = \arg\max_j \frac{\mu_\Delta}{L}$. The definition of function f (Eq.(2)) results in

$$\Big|\frac{\partial f}{\partial \theta_j}\Big|_{\theta=\theta^*}\Big| = \Big|\frac{\sum_{i=1}^n \phi_i\Delta_i \sum_{s=1}^n S(\boldsymbol{x}_s;\boldsymbol{\theta})}{\left(\sum_{i=1}^n S(\boldsymbol{x}_i;\boldsymbol{\theta})\right)^2} - \frac{\sum_{i=1}^n \Delta_i \sum_{s=1}^n \phi_s S(\boldsymbol{x}_s;\boldsymbol{\theta})}{\left(\sum_{i=1}^n S(\boldsymbol{x}_i;\boldsymbol{\theta})\right)^2}\Big|$$
$$= \Big|\frac{\sum_{i=1}^n\sum_{s=1}^n \Delta_i S(\boldsymbol{x}_s;\boldsymbol{\theta})(\phi_i-\phi_s)}{\left(\sum_{i=1}^n S(\boldsymbol{x}_i;\boldsymbol{\theta})\right)^2}\Big|$$

With $\phi_{max} = \max_i|\hat{\phi}(x_i, a_i, y_i)|$, we have,

$$\Big|\frac{\partial f}{\partial \theta_j}\Big|_{\theta=\theta^*}\Big| \le 2\phi_{max}\Big|\frac{\sum_{i=1}^n\sum_{s=1}^n \Delta_i S(\boldsymbol{x}_s;\boldsymbol{\theta})}{\left(\sum_{i=1}^n S(\boldsymbol{x}_i;\boldsymbol{\theta})\right)^2}\Big|$$
$$= 2\phi_{max}\Big|\frac{\sum_{i=1}^n \Delta_i \sum_{s=1}^n S(\boldsymbol{x}_s;\boldsymbol{\theta})}{\left(\sum_{i=1}^n S(\boldsymbol{x}_i;\boldsymbol{\theta})\right)^2}\Big|$$
$$= 2\phi_{max}\frac{\left|\sum_{i=1}^n \Delta_i\right|}{\sum_{i=1}^n S(\boldsymbol{x}_i;\boldsymbol{\theta})} \tag{13}$$

Substituting Eq. (13) into Eq. (12) and taking absolute values, we obtain

$$g(\boldsymbol{\theta}^*) \le \beta\left(\left|\frac{\partial g}{\partial \boldsymbol{\theta}}|_{\theta=\theta^*}\right|\right)^{-1} \frac{2\phi_{max}\left|\sum_{i=1}^n \Delta_i\right|}{\sum_{i=1}^n S(\boldsymbol{x}_i;\boldsymbol{\theta})} \tag{14}$$

Next, we will prove the upper bound of the group size constraint violation and the general linear constraints violation by analyzing $\frac{\partial g}{\partial \boldsymbol{\theta}}|_{\theta=\theta^*}$.

Case 1: $g(\boldsymbol{\theta}^*) = c - \frac{1}{n}\sum_{i=1}^n S(\boldsymbol{x}_i;\boldsymbol{\theta}^*) > 0$: the group size constraint is violated.

$$\frac{\partial g}{\partial \theta_j}|_{\theta=\theta^*} = -\frac{1}{n}\sum_{i=1}^n \Delta_i$$

Substituting this into Eq. (14):

$$g(\boldsymbol{\theta}^*) \le \beta\left(\left|-\frac{1}{n}\sum_{i=1}^n \Delta_i\right|\right)^{-1} \frac{2\phi_{max}\left|\sum_{i=1}^n \Delta_i\right|}{\sum_{i=1}^n S(\boldsymbol{x}_i;\boldsymbol{\theta})}$$
$$= \beta\frac{2\phi_{max}}{\frac{1}{n}\sum_{i=1}^n S(\boldsymbol{x}_i;\boldsymbol{\theta})} \tag{15}$$

Substitute $\beta \le \frac{\xi(c-\xi)\mu_\Delta}{2\phi_{max}L}$ into Eq. (15), we obtain

$$g(\boldsymbol{\theta}^*) \le \frac{\xi(c-\xi)\mu_\Delta}{\frac{1}{n}\sum_{i=1}^n S(\boldsymbol{x}_i;\boldsymbol{\theta})L} \le \frac{\xi(c-\xi)}{\frac{1}{n}\sum_{i=1}^n S(\boldsymbol{x}_i;\boldsymbol{\theta})} = \frac{\xi(c-\xi)}{c-g(\boldsymbol{\theta}^*)}$$

Solving for $g(\boldsymbol{\theta}^*)$, we obtain $g(\boldsymbol{\theta}^*) \le \xi$ (group size constraint satisfied) or $g(\boldsymbol{\theta}^*) \ge c - \xi$ (the model collapses, group size is near zero).

Next, we show that the model is improbable to collapse if the feasible region is non-negligible.

Define the collapsed region as

$$\boldsymbol{\Theta}_{\text{collapse}} := \left\{\boldsymbol{\theta} \in \Theta : \frac{1}{n}\sum_{i=1}^n S(\boldsymbol{x}_i;\boldsymbol{\theta}) < \xi\right\},$$

and the feasible region as

$$\boldsymbol{\Theta}_{\text{feasible}} := \Big\{\boldsymbol{\theta} \in \Theta : \frac{1}{n}\sum_{i=1}^n S(\boldsymbol{x}_i;\boldsymbol{\theta}) \ge c \text{ and other constraints are met}\Big\}$$

for some thresholds $0 < \xi \ll c < 1$. Suppose that the volume of the feasible region dominates that of the collapsed region, i.e.,

$$|\boldsymbol{\Theta}_{\text{collapse}}| \ll |\boldsymbol{\Theta}_{\text{feasible}}|.$$

Given $\forall i, 0 \le S(\boldsymbol{x}_i;\boldsymbol{\theta}) \le 1$, using Hoeffding inequality, we obtain

$$P\left(\sum_{i=1}^n S(\boldsymbol{x}_i;\boldsymbol{\theta}) - \mathbb{E}\sum_{i=1}^n S(\boldsymbol{x}_i;\boldsymbol{\theta}) \le -t\right) \le \exp(-\frac{2t^2}{n}).$$

Let $t = \sqrt{\frac{n\log(1/\delta)}{2}}$, with probability at least $1-\delta$, we have

$$\sum_{i=1}^n S(\boldsymbol{x}_i;\boldsymbol{\theta}) - \mathbb{E}\sum_{i=1}^n S(\boldsymbol{x}_i;\boldsymbol{\theta}) \ge -\sqrt{\frac{n\log(1/\delta)}{2}}$$

Assume $\boldsymbol{\theta}$ is sampled uniformly from $\Theta_{\text{collapse}} \cup \Theta_{\text{feasible}}$, $|\Theta_{collapse}| \ll |\Theta_{feasible}| \Rightarrow P(\boldsymbol{\theta} \in \Theta_{\text{collapse}}) \ll P(\boldsymbol{\theta} \in \Theta_{\text{feasible}})$. Then,

$$\mathbb{E}\sum_{i=1}^{n} S(\boldsymbol{x}_i;\boldsymbol{\theta}) = P(\boldsymbol{\theta} \in \Theta_{collapse}) \cdot \sum_{i=1}^{n} S(\boldsymbol{x}_i;\boldsymbol{\theta}) + P(\boldsymbol{\theta} \in \Theta_{feasible}) \cdot \sum_{i=1}^{n} S(\boldsymbol{x}_i;\boldsymbol{\theta})$$

Given that the contribution from Θ_{collapse} is negligible, we approximate:

$$\mathbb{E}\sum_{i=1}^{n} S(\boldsymbol{x}_i;\boldsymbol{\theta}) \approx \sum_{i=1}^{n} S(\boldsymbol{x}_i;\boldsymbol{\theta}) | \{\boldsymbol{\theta} \in \Theta_{feasible}\} \geq nc$$

Therefore, with probability at least $1-\delta$, we have

$$\begin{aligned}\sum_{i=1}^{n} S(\boldsymbol{x}_i;\boldsymbol{\theta}) &\geq \mathbb{E}\sum_{i=1}^{n} S(\boldsymbol{x}_i;\boldsymbol{\theta}) - \sqrt{\frac{n\log(1/\delta)}{2}} \\ &\geq nc - \sqrt{\frac{n\log(1/\delta)}{2}} \\ \Longrightarrow \frac{1}{n}\sum_{i=1}^{n} S(\boldsymbol{x}_i;\boldsymbol{\theta}) &\geq c - \sqrt{\frac{\log(1/\delta)}{2n}},\end{aligned}$$

i.e. the model is improbable to collapse if the feasible region is non-negligible.

Case 2 : $g(\boldsymbol{\theta}^*) = a^k + \boldsymbol{b}^{k\top} S(\boldsymbol{x};\boldsymbol{\theta}) > 0$:

Similarly, we have

$$g(\boldsymbol{\theta}^*) \leq \beta \left(\left| \sum_{i=1}^{n} b_i^k \Delta_i \right| \right)^{-1} \frac{2\phi_{max} \left| \sum_{i=1}^{n} \Delta_i \right|}{\sum_{i=1}^{n} S(\boldsymbol{x}_i;\boldsymbol{\theta})}. \tag{16}$$

Let $Z = \sum_{i=1}^{n} b_i^k \Delta_i$, then $\mathbb{E}Z = \sum_{i=1}^{n} b_i^k \mathbb{E}\Delta_i = \mu_\Delta \sum_{i=1}^{n} b_i^k$.

By the triangle inequality, we have $\Big||Z| - |\mathbb{E}Z|\Big| \leq |Z - \mathbb{E}Z|$. So, by the Hoeffding inequality, we obtain

$$\begin{aligned}P(\Big||Z| - |\mathbb{E}Z|\Big| \geq t) &\leq P(|Z - \mathbb{E}Z| \geq t) \\ &\leq 2\exp(-\frac{2t^2}{4L^2 \sum_{i=1}^{n} (b_i^k)^2})\end{aligned}$$

Let $t = L\sqrt{2\sum_{i=1}^{n} (b_i^k)^2 \log \frac{2}{\delta}}$. Then with probability at least $1-\delta$,

$$|Z| \geq |\mathbb{E}Z| + t = |\mu_\Delta \sum_{i=1}^{n} b_i^k| + t.$$

Substituting this into Eq. (16), we obtain

$$\begin{aligned}g(\boldsymbol{\theta}^*) &\leq \beta \frac{2\phi_{max} \left| \sum_{i=1}^{n} \Delta_i \right|}{\left(|\mu_\Delta \sum_{i=1}^{n} b_i^k| + t \right) \sum_{i=1}^{n} S(\boldsymbol{x}_i;\boldsymbol{\theta})} \\ &\leq \beta \frac{2\phi_{max} \sum_{i=1}^{n} |\Delta_i|}{\left(|\mu_\Delta \sum_{i=1}^{n} b_i^k| + t \right) \sum_{i=1}^{n} S(\boldsymbol{x}_i;\boldsymbol{\theta})} \\ &\leq \beta \frac{2\phi_{max} L}{\left(|\mu_\Delta \sum_{i=1}^{n} b_i^k| + t \right) \frac{1}{n}\sum_{i=1}^{n} S(\boldsymbol{x}_i;\boldsymbol{\theta})}\end{aligned}$$

As analyzed in **Case 1**, when the feasible region is non-negligible, $\frac{1}{n}\sum_{i=1}^{n} S(\boldsymbol{x}_i;\boldsymbol{\theta}) > c - \xi$ with high probability. Therefore,

$$\begin{aligned}g(\boldsymbol{\theta}^*) &\leq \beta \frac{2\phi_{max} L}{\left(|\mu_\Delta \sum_{i=1}^{n} b_i^k| + t \right)(c-\xi)} \\ &\text{Plug in } \beta \leq \frac{\xi(c-\xi)|\mu_\Delta|}{2\phi_{max} L}, \\ &\leq \frac{\xi|\mu_\Delta|}{|\mu_\Delta \sum_{i=1}^{n} b_i^k| + t} \\ &\leq \frac{\xi|\mu_\Delta|}{|\mu_\Delta \sum_{i=1}^{n} b_i^k| + L\sqrt{2\sum_{i=1}^{n} (b_i^k)^2 \log \frac{2}{\delta}}} \\ &\leq \frac{\xi}{|\sum_{i=1}^{n} b_i^k| + \frac{L}{|\mu_\Delta|}\sqrt{2\sum_{i=1}^{n} (b_i^k)^2 \log \frac{2}{\delta}}}\end{aligned}$$

By Cauchy-Schwarz inequality, we have $\sqrt{\sum_{i=1}^{n} (b_i^k)^2} \geq \frac{|\sum_{i=1}^{n} b_i^k|}{\sqrt{n}}$, therefore,

$$\begin{aligned}g(\boldsymbol{\theta}^*) &\leq \frac{\xi}{|\sum_{i=1}^{n} b_i^k| + \frac{L}{|\mu_\Delta|}\frac{|\sum_{i=1}^{n} b_i^k|}{\sqrt{n}}\sqrt{2\log\frac{2}{\delta}}} \\ &= \frac{\xi}{|\sum_{i=1}^{n} b_i^k| \left(1 + \frac{L}{|\mu_\Delta|\sqrt{n}}\sqrt{\log\frac{2}{\delta}} \right)}\end{aligned}$$

Put together **Case 1** and **Case 2**, we finish the proof.

□

C Implementation Details

C.1 Selection of $\hat{\phi}$

$\frac{\sum_{i=1}^{n} S(\boldsymbol{x}_i;\theta)\hat{\phi}(\boldsymbol{x}_i,a_i,y_i)}{\sum_{i=1}^{n} S(\boldsymbol{x}_i;\theta)}$ can be used to represent the ATE estimated by different methods. And the following $\hat{\phi}_{\text{iptw}}$ and $\hat{\phi}_{\text{aiptw}}$, correspond to the ATE estimated using IPTW and AIPTW, respectively. *inverse probability of treatment weighting* (IPTW) and *augmented inverse probability of treatment weighting* (AIPTW), respectively. To clarify, $\phi(\boldsymbol{x}_i, a_i, y_i)$ does not depend on the parameter $\boldsymbol{\theta}$, these estimates are separated from the parametric surrogate model S.

$$\begin{aligned}\hat{\phi}_{\text{iptw}}(\boldsymbol{x}_i, a_i, y_i) =& \frac{a_i}{\hat{e}(\boldsymbol{x}_i)} y_i - \frac{1-a_i}{1-\hat{e}(\boldsymbol{x}_i)} y_i, \\ \hat{\phi}_{\text{aiptw}}(\boldsymbol{x}_i, a_i, y_i) =& \hat{\mu}_1(\boldsymbol{x}_i) - \hat{\mu}_0(\boldsymbol{x}_i) + \frac{a_i}{\hat{e}(\boldsymbol{x}_i)}(y_i - \hat{\mu}_1(\boldsymbol{x}_i)) \\ &- \frac{1-a_i}{1-\hat{e}(\boldsymbol{x}_i)}(y_i - \hat{\mu}_0(\boldsymbol{x}_i)).\end{aligned}$$

C.2 Synthetic Data Generation

Let $\sigma_X, \sigma_Y, \rho \in \mathbb{R}$ be fixed constants, and let $\beta_1, \beta_\tau, \omega \in \mathbb{R}^p$. Draw $\boldsymbol{X}$ according to a multi-variate normal distribution, and $A, Y(0), Y(1)$ as follows:

$$\boldsymbol{X} \sim \mathcal{MVN}(0, \sigma_X^2[(1-\rho)I_p + \rho 1_p 1_p^T]), \tag{17}$$

$$A|\boldsymbol{X} \sim \text{Bernoulli}(\sigma(\boldsymbol{X}^T\omega)), \tag{18}$$

$$\epsilon \sim \mathcal{N}(0, \sigma_Y^2), \tag{19}$$

$$Y(0) = (\sin(10 * \boldsymbol{X}) + 5 * \boldsymbol{X}^2)^T \beta_1 + \epsilon, \tag{20}$$

$$Y(1) = (\sin(10 * \boldsymbol{X}) + 5 * \boldsymbol{X}^2)^T \beta_1 + \boldsymbol{X}^T \beta_\tau + \epsilon. \tag{21}$$

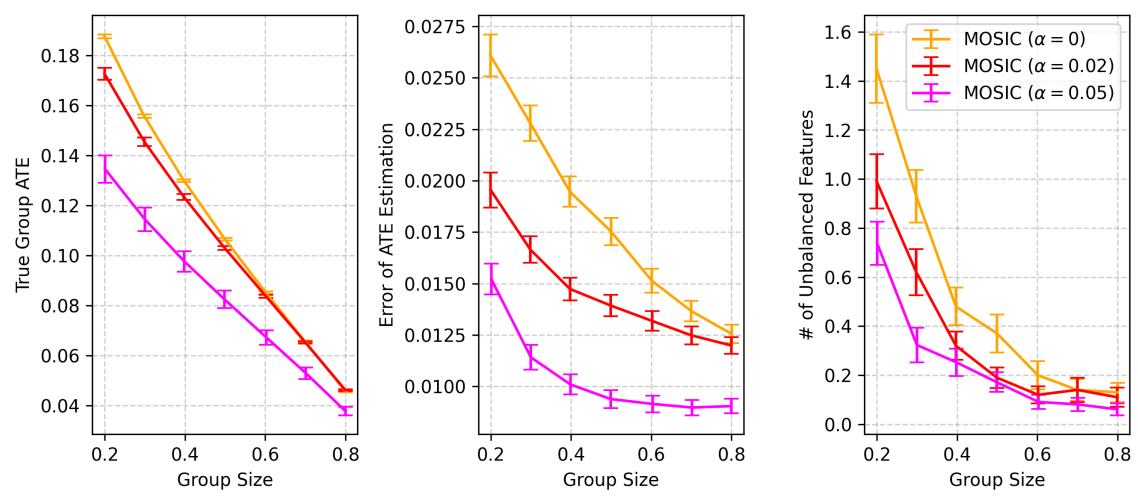

Figure E.1: Correlation of ATE estimation error and the number of unbalanced features on confounded synthetic dataset ($\tilde{\omega} = 5$), obtained using MOSIC with varying α.

Dataset	Dragonnet	LR
Synthetic	0.10	0.10
MIMIC-IV	2.32	1.57
eICU	1.08	1.10

Table C.1: Number of unbalanced features after reweighting by propensity scores obtained by propensity models

We set the parameters as follows:

$$p = 10, \quad \sigma_X = \sigma_Y = 0.1, \quad \rho = 0.3,$$
$$\beta_1 = [0, 0, 0, 0, 2, 0, 0, 0, 0, 0],$$
$$\beta_\tau = [0.5, 0.5, 0.5, 0.5, 0, 0, 0, 0, 0, 0],$$
$$\omega = [0, -1 \cdot \tilde{\omega}, -1 \cdot \tilde{\omega}, 1 \cdot \tilde{\omega}, 1 \cdot \tilde{\omega}, -2 \cdot \tilde{\omega}, 0, 0, 0, 0].$$

The *imbalance parameter*, $\tilde{\omega} \geq 0$, scales the magnitude of the treatment assignment weight vector ω. In particular, we generated two synthetic datasets: (1) no confounding bias ($\tilde{\omega} = 0$); and (2) high confounding bias ($\tilde{\omega} = 5$). As there is no limitation to generate synthetic data, we set the total sample size for synthetic data to 5,000 to study the model performance under finite samples and use a balanced 50/50 train-test split. As for real-world datasets, we use a 70/30 train-test split.

C.3 Baseline Implementation

CAPITAL identifies a subgroup by maximizing its size while ensuring the CATE exceeds a predefined threshold. Since subgroup size is not directly controlled in this setting, we vary the CATE threshold and construct the group size vs. ATE curve for comparison.
OWL is originally designed for individual treatment rule estimation but can be viewed as a subgroup identification method without interpretability constraints. It assigns scores between 0 and 1, which we threshold to obtain subgroups of a desired size. We implement OWL using the DTRlearn2 R package.
VT The original VT supports both binary and continuous outcomes, but the commonly used R package aVirtualTwins handles only binary outcomes. Since our experiments require both, we implemented our own VT following [20]: first estimating treatment effects with a random forest, and then fitting a regression tree to assign subgroup scores. Subgroups of different sizes are obtained by thresholding these scores.
Dragonnet & CT & CF We adopt the implementations from the Python package causalml.

C.4 Model Selection for Nuisance Function Estimation

We consider LR and Dragonnet for estimating the propensity score model. We follow the literature [58] to use the number of unbalanced features after inverse propensity score weighting as the metric (the lower the better) to select the propensity score model. The results are shown in Table C.1, which shows that LR performs as well as or better than Dragonnet. Thus we select LR as the propensity score model for all datasets.

For the potential outcome model, we consider Dragonnet, CF, and CT, as they are the CATE estimators we are comparing against in subgroup identification. Theoretically, CF improves upon CT by reducing bias and producing smoother decision boundaries through aggregating CTs. As for Dragonnet and CF, prior work [30] has shown that Dragonnet outperforms CF.

While many other methods exist for nuisance function estimation, our goal is to demonstrate MOSIC's flexibility rather than prescribe a specific estimator. If more effective models are developed or if a particular method performs better in a given setting, we encourage adapting those for nuisance function estimation.

C.5 Hyperparameter Tuning

CAPITAL optimizes the policy tree depth with $max\_depth \in \{2, 3\}$. VT selects decision tree depth for subgroup identification from $max\_depth \in \{3, 5, 7, 10\}$. Similarly, CT uses $max\_depth \in \{3, 5, 7, 10\}$, while CF considers both tree depth $max\_depth \in \{3, 5, 7, 10\}$ and the number of trees $num\_tree \in \{5, 10, 20, 50, 100\}$. Moreover, Dragonnet tunes the hidden layer size with $hidden\_size \in \{50, 100, 200\}$. Finally, the hyperparameters for MOSIC include $\beta \in \{10^{-2}, 10^{-3}, 10^{-4}, 10^{-5}\}$ and those depending on the implementation of the subgroup identification model. For MOSIC-MLP, we tune the hidden layer size with $hidden\_size \in \{50, 100, 200\}$.

D Real-world observational study setup

We provide details of our real-world observational study setup using the target trial emulation framework in Table D.1 and Table D.2.

E Additional Experiment Results

E.1 Correlation of ATE estimation error and feature imbalance

Figure E.1 numerically verifies that a higher number of unbalanced features is correlated with larger estimation error, justifying feature imbalance as a proxy for estimation reliability in real-world experiments. Further, a higher overlap threshold α reduces the subgroup ATE estimation error, suggesting that enforcing stronger overlap improves estimation reliability.

E.2 Significance Tests on Real-world data

Table E.1 presents the p-values comparing MOSIC ($\alpha = 0.01$) against baseline methods, corresponding to the hypothesis test that MOSIC differs from the baselines. These results directly support the performance trends shown in Figure 2. For instance, in eICU experiments with c=0.5:

Table D.1: Target Trial Emulation for 7-day mortality

Protocol component	Target trial specification	Target trial emulation
Enrollment window	Within 24 hours after ICU admission	Within 24 hours after ICU admission
Eligibility criteria	Adult; Patient has sepsis at enrollment window; No sepsis history before enrollment window; No corticosteroid prescription more than 10 hrs before enrollment window	Adult; Patients meet Sepsis-3 criteria at enrollment window; No sepsis history before enrollment window; No corticosteroid prescription more than 10 hrs before enrollment window
Treatment strategies	Treated: Initiation of hydrocortisone at a dose of 160 mg at enrollment window;<br><br>Control: No initiation of any corticosteroid drug at enrollment window	Treated = receiving >= 160mg per day hydrocortisone-equivalent corticosteroid;<br><br>Control = Otherwise.<br><br>We consider prednisolone, prednisone, hydrocortisone, dexamethasone, and methylprednisolone.<br>Conversions:<br>1 mg Prednisolone = 4 mg Hydrocortisone<br>1 mg Prednisone = 4 mg Hydrocortisone<br>1 mg Dexamethasone = 25 mg Hydrocortisone<br>1 mg Methylprednisolone = 5 mg Hydrocortisone
Treatment assignment	Patients are randomly assigned to either treatment strategy on the first day after ICU admission and are aware of the strategy they are assigned to.	Randomization is emulated at enrollment by adjustment for baseline confounders.<br><br>Covariates include Demographics (Age, gender); Labs/vitals (GCS, Lactate, MAP, PaO2, Platelet, Respiratory rate, Systolic ABP, Temperature, Urine, WBC); Comorbidity (Elixhauser Comorbidity Index, chronic lung disease, liver disease, AIDS); SOFA score and its organ-specific subscores.
Follow-up	We follow each patient from their baseline until their death, loss of follow-up, or discharge, whichever occurs first.	We follow each patient from their baseline until their death, loss of follow-up, or discharge, whichever occurs first.
Causal contrasts	Intention-to-treat effect.	Observational analog of intention-to-treat effect.
Statistical analysis	Mean difference in 7-day mortality	Mean difference in 7-day mortality estimated using AIPW with both outcome and propensity models.

- MOSIC achieves significantly better ATE than Dragonnet (p=0.0057)
- MOSIC maintains significantly fewer unbalanced features than Dragonnet (p=9.2E-04)

Table D.2: Target Trial Emulation for ventilation-free days within 7 days

Protocol component	Target trial specification	Target trial emulation
Enrollment window	Within 24 hours after ICU admission	Within 24 hours after ICU admission
Eligibility criteria	Adult; Patient has sepsis at enrollment window; No sepsis history before enrollment window; No corticosteroid prescription more than 10 hrs before enrollment window	Adult; Patients meet Sepsis-3 criteria at enrollment window; No sepsis history before enrollment window; No corticosteroid prescription more than 10 hrs before enrollment window
Treatment strategies	Treated: Initiation of hydrocortisone at a dose of 160 mg at enrollment window;<br><br>Control: No initiation of any corticosteroid drug at enrollment window	Treated = receiving >= 160mg per day hydrocortisone-equivalent corticosteroid;<br><br>Control = Otherwise.<br><br>We consider prednisolone, prednisone, hydrocortisone, dexamethasone, and methylprednisolone.<br>Conversions:<br>1 mg Prednisolone = 4 mg Hydrocortisone<br>1 mg Prednisone = 4 mg Hydrocortisone<br>1 mg Dexamethasone = 25 mg Hydrocortisone<br>1 mg Methylprednisolone = 5 mg Hydrocortisone
Treatment assignment	Patients are randomly assigned to either treatment strategy on the first day after ICU admission and are aware of the strategy they are assigned to.	Randomization is emulated at enrollment by adjustment for baseline confounders.<br><br>Covariates include Demographics (Age, gender); Labs/vitals (GCS, Lactate, MAP, PaO2, Platelet, Respiratory rate, Systolic ABP, Temperature, Urine, WBC); Comorbidity (Elixhauser Comorbidity Index, chronic lung disease, liver disease, AIDS); SOFA score and its organ-specific subscores.
Follow-up	We follow each patient from their baseline until their death, loss of follow-up, or discharge, whichever occurs first.	We follow each patient from their baseline until their death, loss of follow-up, or discharge, whichever occurs first.
Causal contrasts	Intention-to-treat effect.	Observational analog of intention-to-treat effect.
Statistical analysis	Mean difference in ventilation-free days within 7 days	Mean difference in ventilation-free days within 7 days estimated using AIPW with both outcome and propensity models.

E.3 Uncertainty quantification

To quantify uncertainty, we computed the asymptotic 95% confidence intervals based on the closed-form influence function of the AIPTW estimator. Figure E.2 reports the distribution of CI widths

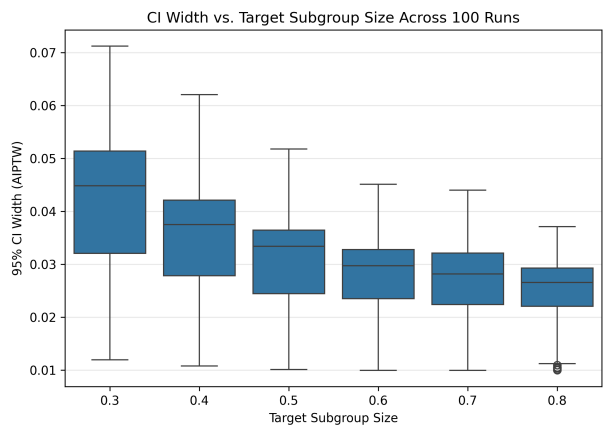

(a) Unconfounded

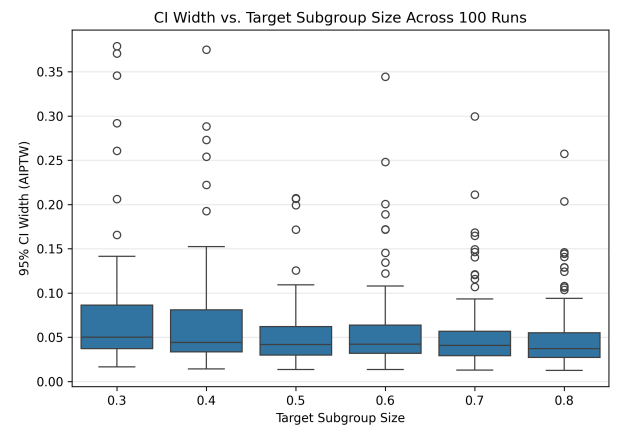

(b) Confounded

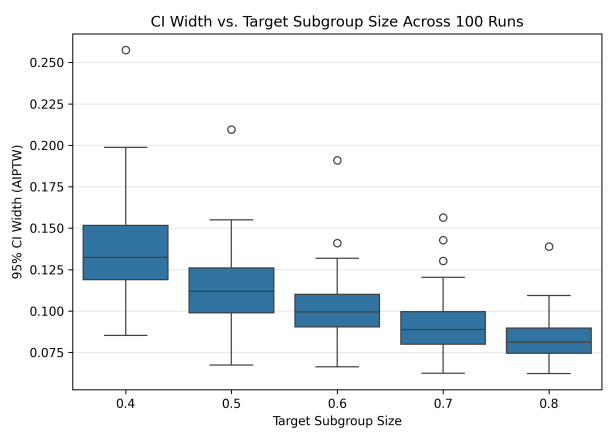

(c) eICU

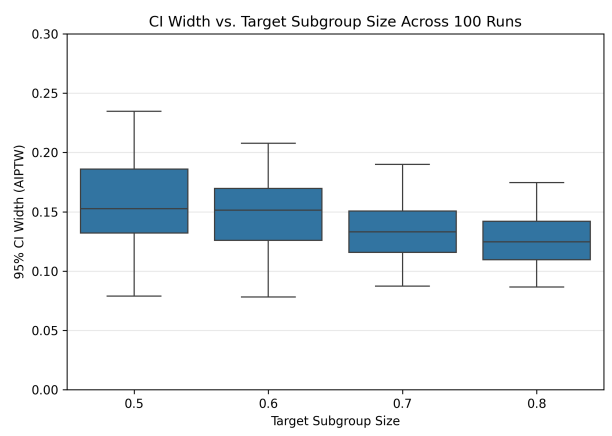

(d) MIMIC-IV

Figure E.2: CI width.

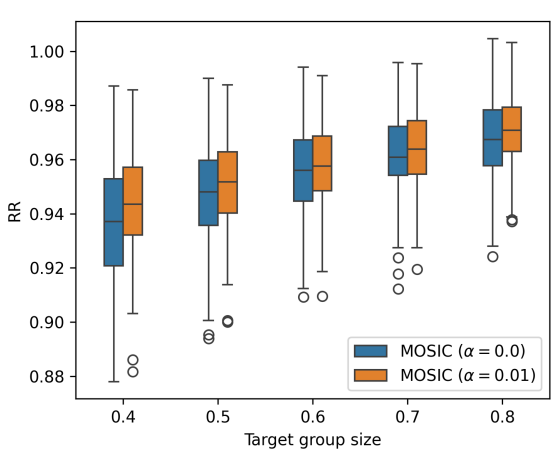

(a) Risk Ratio

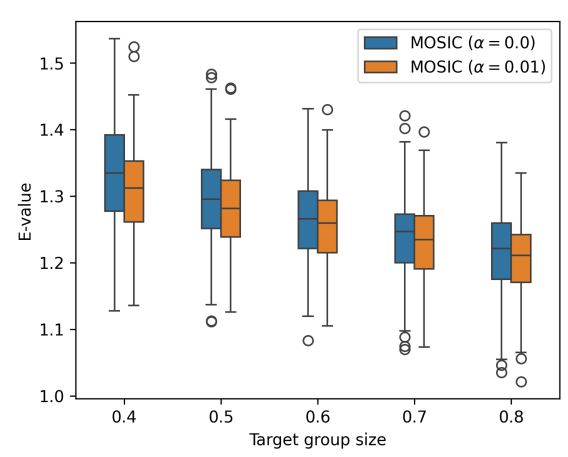

(b) E-value

Figure E.3: Sensitivity analysis on unmeasured confounding

across 100 independent train–test splits, showing the anticipated increase in uncertainty as subgroup size decreases.

Additionally, we report the Risk Ratio (RR) and E-values to measure the robustness of our subgroup results with respect to unmeasured confounders.

E.4 Additional Baselines

We further compare against DR-learner, R-learner, BART, and an overlap-weighted variant of MOSIC (MOSIC-OW). We adopted the Python package causalml to implement DR-learner and R-learner, using Random Forest as their base learner. For BART, we adopted the R package bartCause. For hyperparameters, DR-learner and R-learner consider $max\_depth \in \{3, 5, 7, 10\}$, and BART considers the number of prior standard deviations $k \in \{1, 2, 3\}$. The hyperparameter tuning procedures are the same as described in Section 5.1.

E.5 Overlap constraint on synthetic data

We numerically verify that MOSIC can indeed accommodate the overlap constraint (Figure E.6). Without overlap constraints, MOSIC selects patients with the highest ITEs, including those in the non-overlap region. With overlap constraints (Figure E.6, left), MOSIC continues to select patients with large ITEs but systematically excludes those who violate the overlap constraint ($\hat{e}(x) < 0.05$ or $\hat{e}(x) > 0.95$).

E.6 Test Set Overlap on Real-world Data

We evaluated overlap on the held-out test sets and report the proportions of samples with estimated propensity scores falling outside [0.01,0.99], [0.02,0.98], and [0.05,0.95] within the subgroups selected on the test sets (Figure E.5 and 3). When no overlap constraint is imposed ($\alpha = 0$), the proportion of low-overlap samples in the selected subgroup closely matches that of the full test set. In contrast, when overlap constraints are activated ($\alpha > 0$), these proportions decrease substantially across all thresholds, demonstrating that MOSIC's overlap constraint effectively improves overlap in the selected subgroups during evaluation as well as training.

E.7 # Unbalanced features on real-world data using SMD>0.1 as threshold

SMD>0.1 is a stricter threshold to evaluate feature imbalance and commonly used in epidemiology studies. However, Austin (2009) notes that "For modest sample sizes, one could expect standardized differences that exceed 0.20 (20 percent) even when the propensity-score model was correctly specified." Given that both of our real-world cohorts are relatively small (13,361 patients in eICU and 6,516 in MIMIC), we chose SMD > 0.2 as the primary threshold to reduce the risk of flagging spurious imbalance driven by limited sample size. This threshold is also frequently used in epidemiology studies.

We now report the results using the stricter SMD > 0.1 criterion, and it does not alter our main conclusions. Although the absolute number of unbalanced covariates increases across all methods due to inherent data size limitations, the relative comparison remains the same: MOSIC achieves comparable balance while yielding substantially higher subgroup ATEs (Figure E.7, Table E.2 and Figure 2). (While Dragonnet demonstrates significantly lower feature imbalance, our method obtains significantly higher subgroup ATE improvement than Dragonnet, as shown in Figure 2b.)

E.8 Additional Balance Diagnostics

To further evaluate the reliability of the identified subgroups, we report three additional diagnostics on the alternative outcome setting, ventilation-free days within 7 days of ICU admission: effective sample size (ESS), the proportion of samples with propensity scores outside ([0.05,0.95]) (% Extreme), and the maximum inverse propensity weight (Max IPW). Consistent with the subgroup ATE and feature-balance results reported in Table 1, MOSIC achieves higher ESS, lower proportions of extreme propensity scores, and smaller maximum inverse propensity weights across subgroup sizes. These complementary metrics further support that MOSIC identifies subgroups with improved overlap and more reliable treatment-effect estimation.

E.9 Extension to Safety, Budget, and Fairness Constraint on Synthetic Data

To demonstrate MOSIC's flexibility to handle additional constraints, we extend the synthetic data generation process described in Appendix C.2 by introducing a safety, budget, and fairness constraint. In particular:

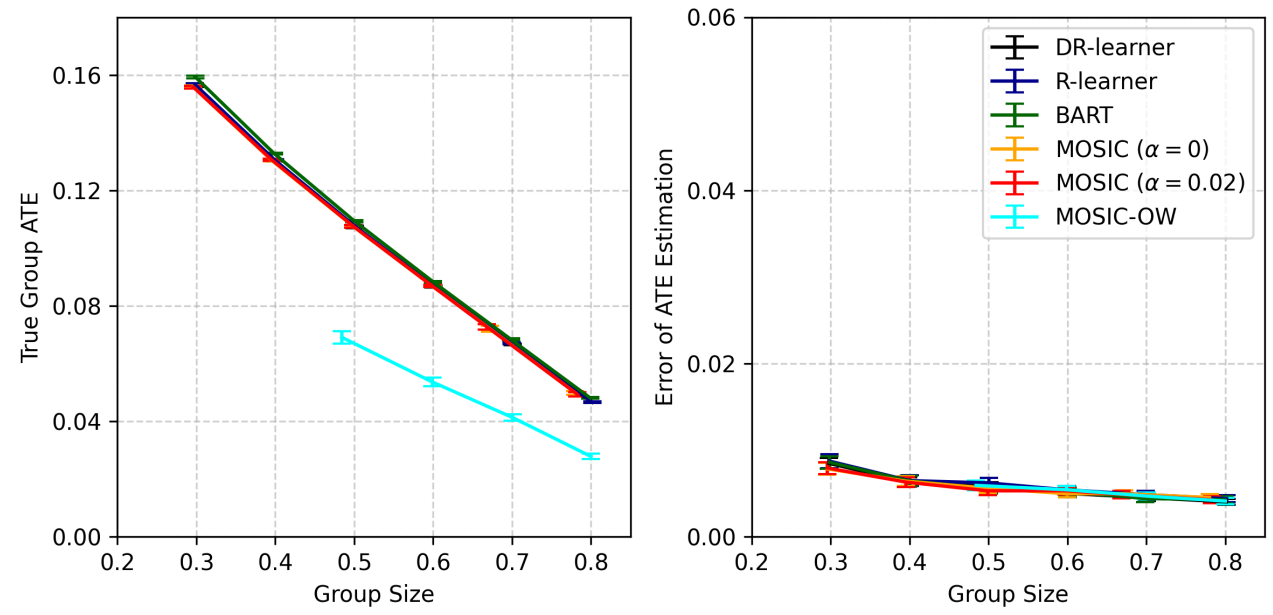

(a) Unconfounded($\tilde{\omega} = 0$)

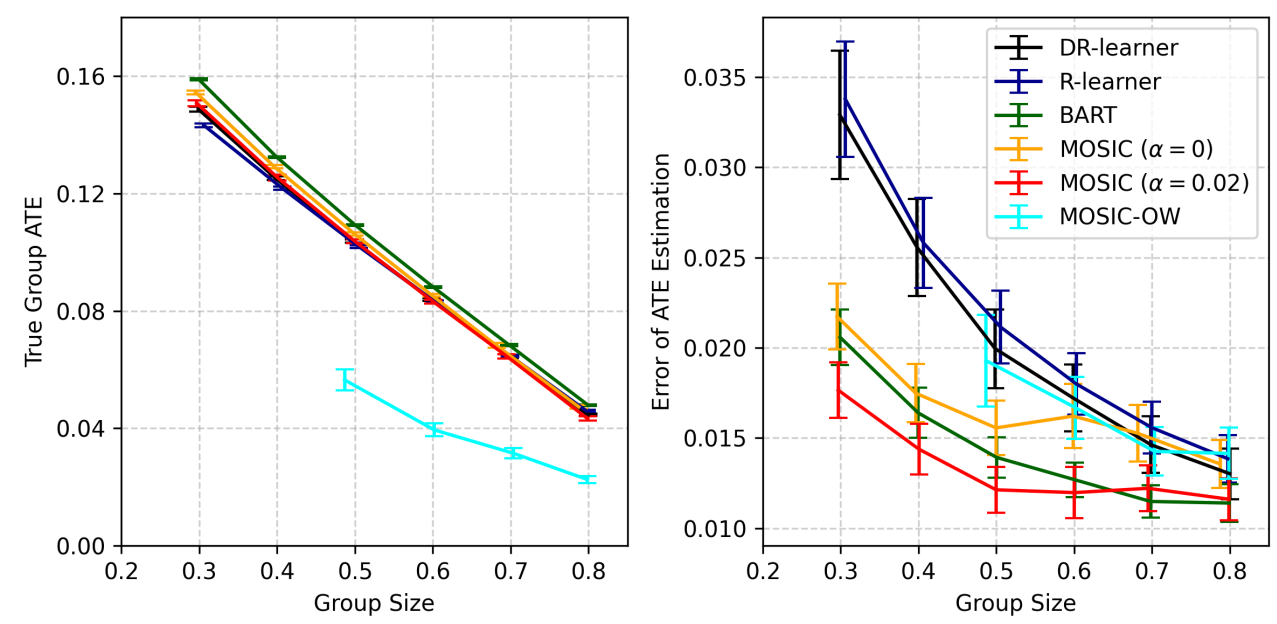

(b) Confounded($\tilde{\omega} = 5$)

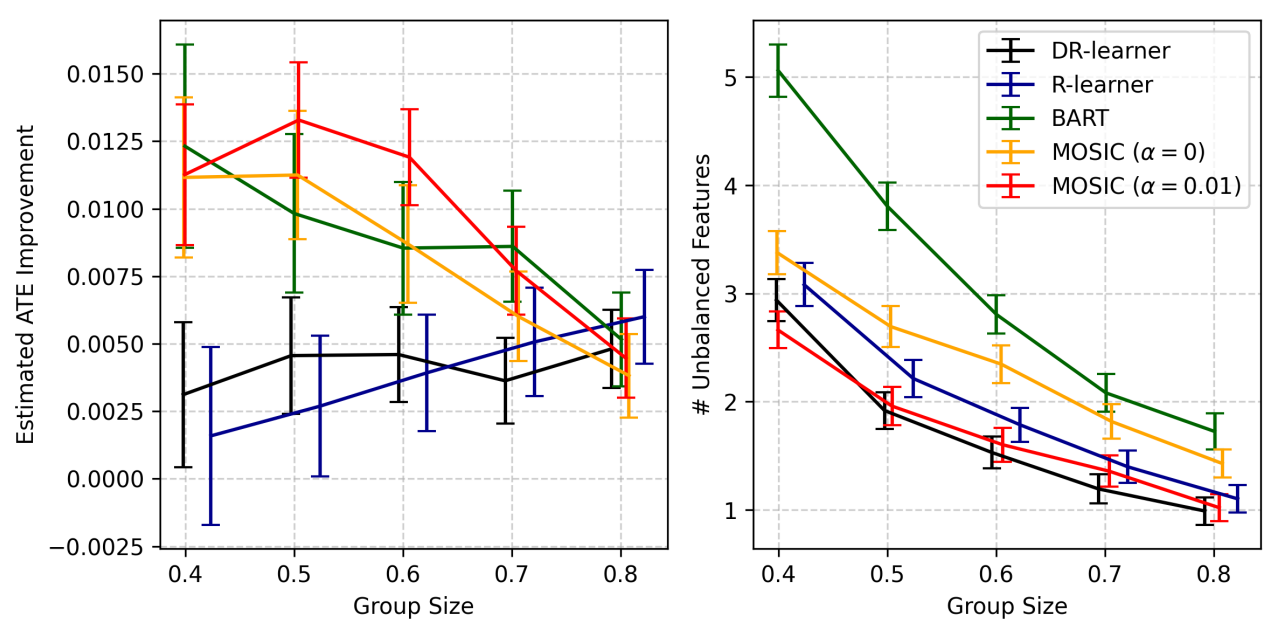

(c) eICU

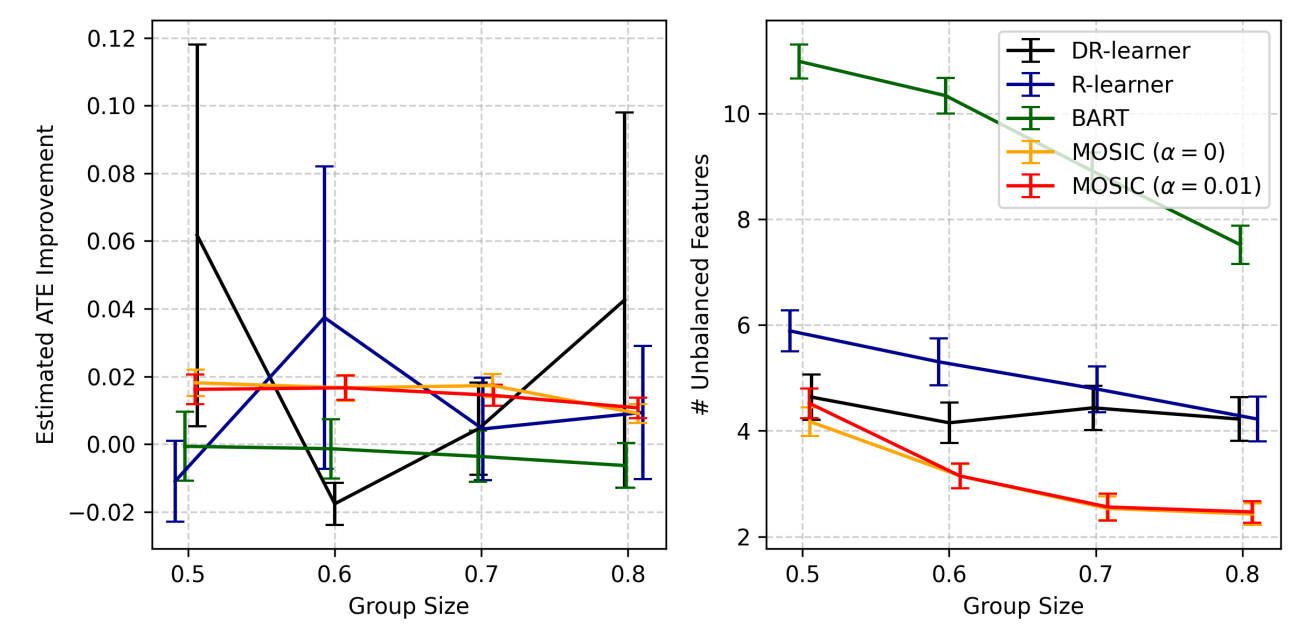

(d) MIMIC-IV

Figure E.4: Estimated ATE and the number of unbalanced features on real-world datasets. MOSIC-OW performs substantially worse in synthetic experiments, so we omit it from real-world comparisons.

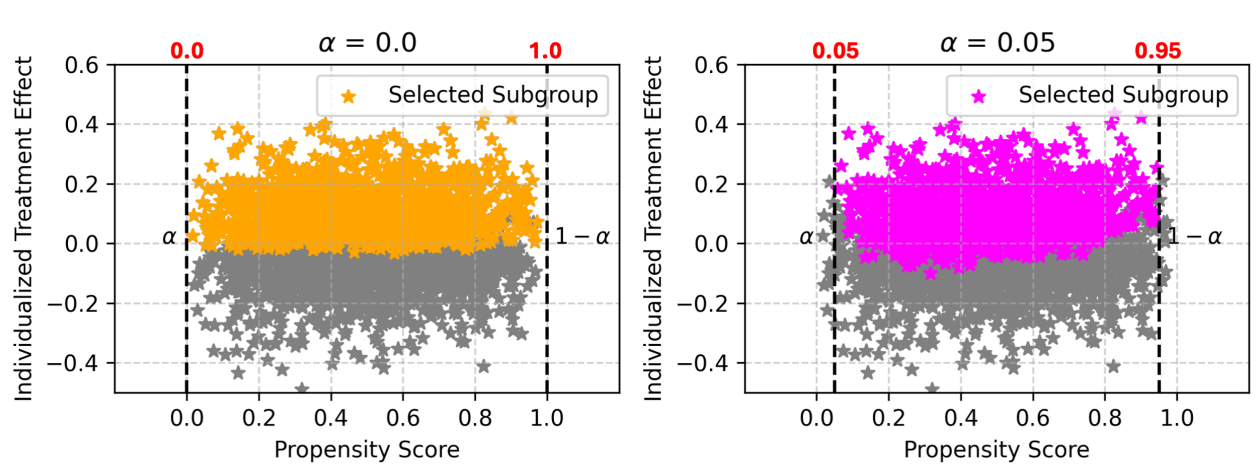

Figure E.6: Results on synthetic data with confounding bias ($\tilde{\omega}$ = 5). Each dot represents an individual, with the x-axis denoting the estimated propensity score, the y-axis representing the true individual treatment effect (ITE), and the vertical lines indicating the desired overlap threshold.

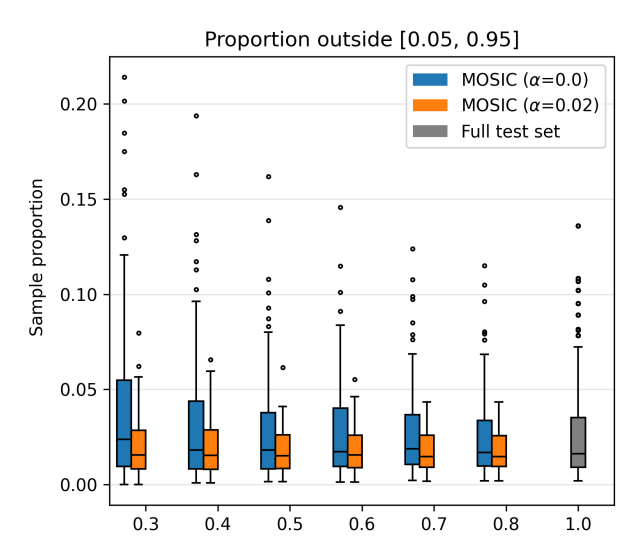

(a) Confounded Synthetic Data($\tilde{\omega} = 5$)

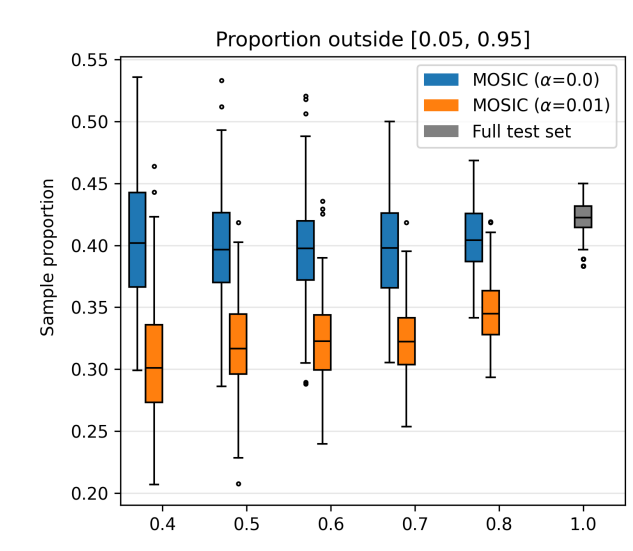

(b) eICU

Figure E.5: Overlap Evaluation on Test Set.

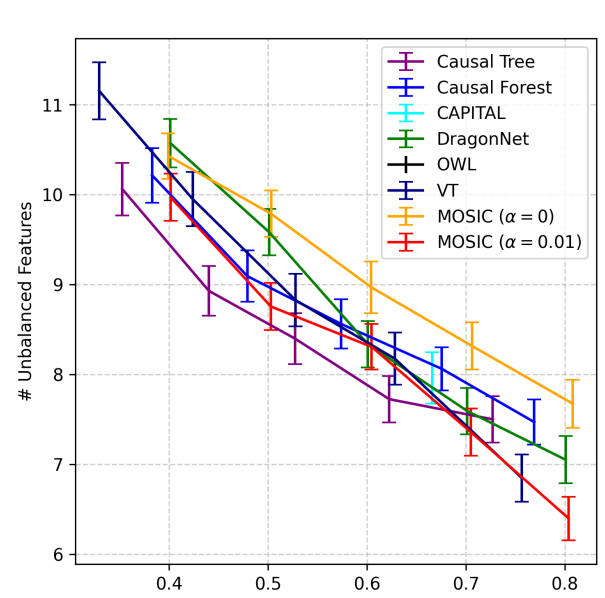

(a) eICU

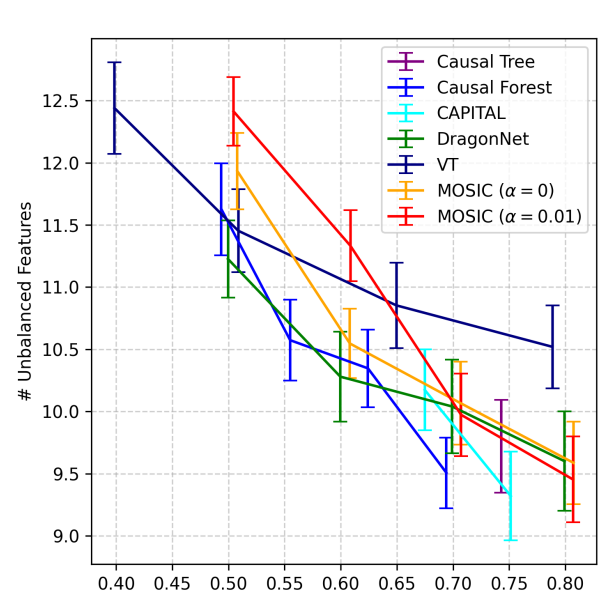

(b) MIMIC

Figure E.7: Number of unbalanced features using SMD>0.1 as threshold.

- Safety constraint: Following Doubleday et al. [18], each sample is assigned a risk score $r_i = 1/(1 + \exp(10 * x_i[10] + 1))$. We require the average risk of the selected subgroup to be no greater than 0.05:

$$\frac{\sum_{i=1}^{n} \mathbb{1}(S(x_i) > 0.5) r_i}{\sum_{i=1}^{n} \mathbb{1}(S(x_i) > 0.5)} \leq 0.05.$$

Table E.1: P-values for ATE and feature balance comparisons between MOSIC ($\alpha = 0.01$) and baseline methods.

		$c = 0.4$	$c = 0.5$	$c = 0.6$	$c = 0.7$	$c = 0.8$
			**eICU**			
ATE	CT	0.71	0.065	0.0011	0.0046	0.025
	CF	0.19	0.0048	0.00019	0.0012	0.0057
	CAPITAL	-	-	-	3.5E-05	-
	Dragonnet	0.30	0.0057	5.5E-04	0.011	0.11
	OWL	0.27	0.0019	0.0010	0.084	0.16
	VT	0.38	0.07	0.039	0.31	0.46
Balance	CT	0.16	0.58	0.56	0.65	0.04
	CF	0.35	0.12	0.48	0.47	0.16
	CAPITAL	-	-	-	0.59	-
	Dragonnet	0.022	9.2E-04	0.14	0.33	0.066
	OWL	0.0088	0.0077	0.13	0.22	0.046
	VT	0.0011	0.0060	0.036	0.23	0.39
			**MIMIC-IV**			
ATE	CT	-	-	-	-	0.02
	CF	-	0.42	0.68	0.35	0.16
	CAPITAL	-	0.021	-	0.30	0.72
	Dragonnet	-	0.039	0.10	0.21	0.30
	VT	-	0.10	0.16	0.15	0.15
Balance	CT	-	-	-	-	0.63
	CF	-	0.19	5.1E-04	1.3E-04	1.1E-04
	CAPITAL	-	6.3E-07	-	0.17	3.3E-05
	Dragonnet	-	0.062	0.80	0.28	0.48
	VT	-	0.033	0.0020	0.018	0.20

Table E.2: P-values for feature balance comparisons between MOSIC and baseline methods using SMD> 0.1 as the threshold for an unbalanced feature.

	$c = 0.4$	$c = 0.5$	$c = 0.6$	$c = 0.7$	$c = 0.8$
		**eICU**			
CAPITAL	–	–	0.36	–	–
CF	0.54	0.39	0.50	0.050	0.0024
CT	0.82	0.65	0.81	0.32	0.0022
Dragonnet	0.11	0.027	0.93	0.52	0.069
VT	0.0044	0.0033	0.18	0.04	0.21
		**MIMIC-IV**			
CAPITAL	–	1.8E-10	–	0.67	–
CF	–	0.091	0.081	0.41	0.91
CT	–	–	–	–	0.60
Dragonnet	–	0.0049	0.024	0.89	0.78
VT	–	0.095	0.79	0.067	0.028

- Budget constraint: Following Qiu et al. [44], each sample is assigned a treatment cost value $cost_i = (x_i[3] + 5)/5$. The total cost of the selected subgroup must not exceed half the cost of treating the entire population (assuming a unit cost per sample):
$$\sum_{i=1}^{n} \mathbb{1}(S(x_i) > 0.5)cost_i \leq 0.5 * n.$$
The test contains 2500 samples, so the total cost limit $0.5*n = 1250$ in this case.
- Fairness constraint: Let a binary sensitive attribute: $sens_i = \mathbb{1}(x[3] > 0.5)$. We adopt the conditional statistical parity metric [39]. In our setting, this corresponds to maintaining a sensitive-group proportion of 0.5 in the selected subgroup:
$$\left| \frac{\sum_{i=1}^{n} \mathbb{1}(S(x_i) > 0.5)sens_i}{\sum_{i=1}^{n} \mathbb{1}(S(x_i) > 0.5)} - 0.5 \right| \leq 0.01,$$
where we allow a violation of 0.01. We can then convert this to two ratio-form constraints:
$$-0.01 \leq \frac{\sum_{i=1}^{n} \mathbb{1}(S(x_i) > 0.5)sens_i}{\sum_{i=1}^{n} \mathbb{1}(S(x_i) > 0.5)} - 0.5 \leq 0.01.$$

In addition to the group size constraint ($c = 0.5$) and overlap constraint ($\alpha = 0.02$), we progressively add the following constraints: 1) Plus safety constraint; 2) Plus safety and budget constraint; 3) Plus safety, budget, and fairness constraint. Results in Table 2 show that MOSIC can effectively enforce all of these constraints.

E.10 Extension to Safety Constraint on eICU

To impose the interpretability requirement, we implement MOSIC-DT for this setting. Similarly, we run experiments on 100 random train-test splits and use 5-fold cross-validation to select the tree depth among {3,5,7}. Results in Table 3 show MOSIC can effectively exclude patients with GCS< 6. Additionally, an example (Figure 6(b)) shows that MOSIC indeed learned to exclude such high-risk patients.

We additionally run MOSIC-MLP with and without the GCS< 6 constraints and run post-hoc SHAP-value analysis to evaluate the feature contributions. The dominant features identified by SHAP are generally consistent with the rule structure revealed by the decision tree. In particular, the SHAP values of the GCS features were ambiguous before we enforced the constraint that avoids patients with GCS < 6, but they became clearly separated after we enforced it, aligning well with our intention.

We further evaluate subgroup stability across different initializations. For the same train–test split, we reran MOSIC-MLP six times with the same constraints but different random initializations and examined whether the resulting feature contributions remained consistent. As shown in Figure E.8, the SHAP value patterns are highly similar across runs, suggesting that the learned subgroups are stable with respect to initialization and all align well with the safety constraint.

F Evaluation on Binary Subgroup Setting

Unlike approaches that assume the existence of two or a finite number of subgroups with distinct ATEs, our study focuses on identifying a subset of the population with maximal ATE under real-world constraints, without making structural assumptions about the underlying heterogeneity. This design enables our method to generalize to continuous or complex heterogeneity. Nevertheless, we recognize that such structural assumptions, such as that binary subgroups with distinct ATE exist, are plausible in certain real-world scenarios. In such cases, the real-world constraint will naturally introduce a trade-off for identification performance.

To illustrate, we modify the DGP of synthetic data by replacing $Y(1)$ in Appendix C.2 to:
$$Y(1) = (\sin(10 * \boldsymbol{X}) + 5 * \boldsymbol{X}^2)^T \beta_1 + \mathbb{1}(\boldsymbol{X}^T > 0.05)\beta_\tau + \epsilon.$$

Table E.3: Additional reliability diagnostics on the ventilation-free-days outcome. Higher ESS and lower % Extreme / Max IPW indicate better overlap.

Method	ESS			% Extreme			Max IPW		
	$c = 0.6$	$c = 0.7$	$c = 0.8$	$c = 0.6$	$c = 0.7$	$c = 0.8$	$c = 0.6$	$c = 0.7$	$c = 0.8$
OWL	347.27±5.03	401.11±5.35	454.09±5.92	0.24±0.004	0.24±0.004	0.25±0.003	45.88±1.68	47.80±1.79	48.70±1.77
VT	327.13±4.21	378.74±5.07	430.49±5.43	0.25±0.003	0.25±0.003	0.25±0.003	44.97±1.32	47.04±1.61	49.11±1.73
CT	298.06±4.74	360.14±5.27	422.01±5.46	0.24±0.004	0.24±0.003	0.25±0.003	41.74±1.27	43.79±1.30	46.58±1.45
CF	342.29±4.96	392.13±5.37	441.76±5.75	0.23±0.003	0.24±0.003	0.25±0.003	45.13±1.47	46.71±1.44	49.26±1.74
Dragonnet	332.98±4.72	388.79±5.22	441.91±5.44	0.28±0.00	0.27±0.00	0.27±0.00	46.53±1.47	47.49±1.44	48.07±1.41
MOSIC-MLP ($\alpha = 0.01$)	384.80±5.63	438.94±5.55	488.80±5.55	0.20±0.003	0.20±0.003	0.22±0.002	43.30±1.75	44.88±1.70	46.08±1.09
MOSIC-DT ($\alpha = 0.01$)	459.98±5.54	500.53±6.27	518.43±6.14	0.19±0.003	0.20±0.004	0.21±0.003	40.53±0.87	47.49±1.44	48.07±1.41
MOSIC-Forest ($\alpha = 0.01$)	489.73±4.68	520.97±5.87	531.92±5.79	0.17±0.003	0.18±0.004	0.20±0.004	45.13±1.47	46.71±1.44	49.26±1.74

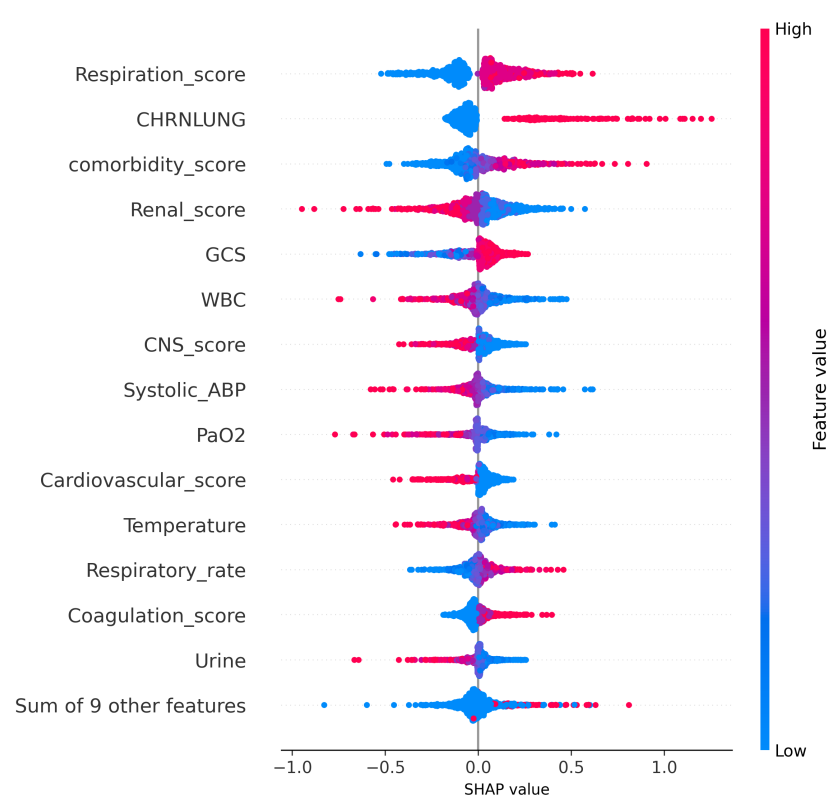

(a) Seed 1

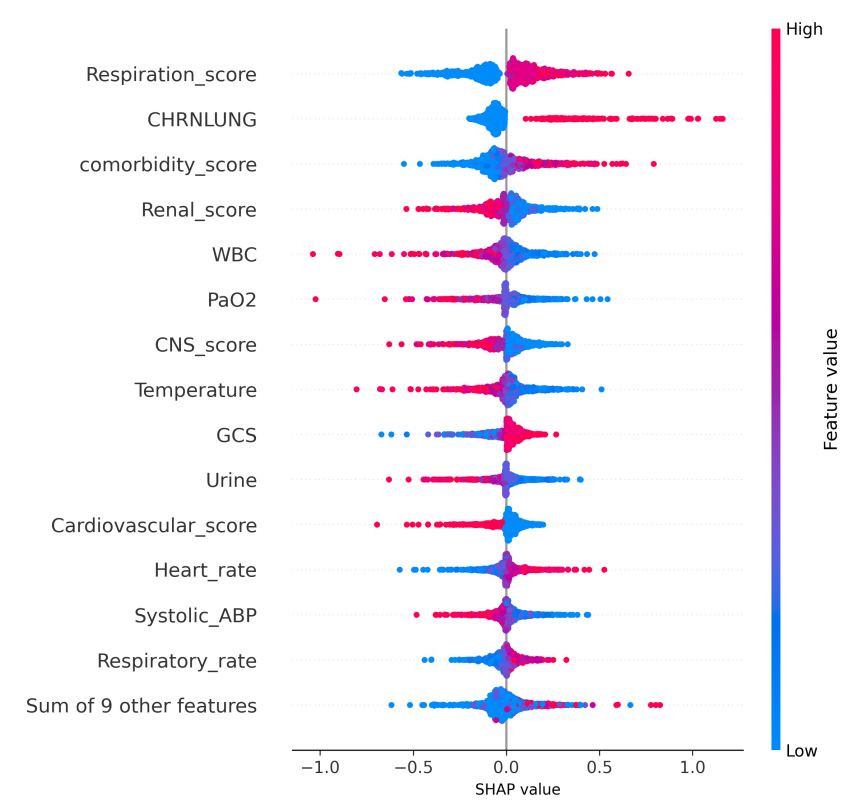

(b) Seed 2

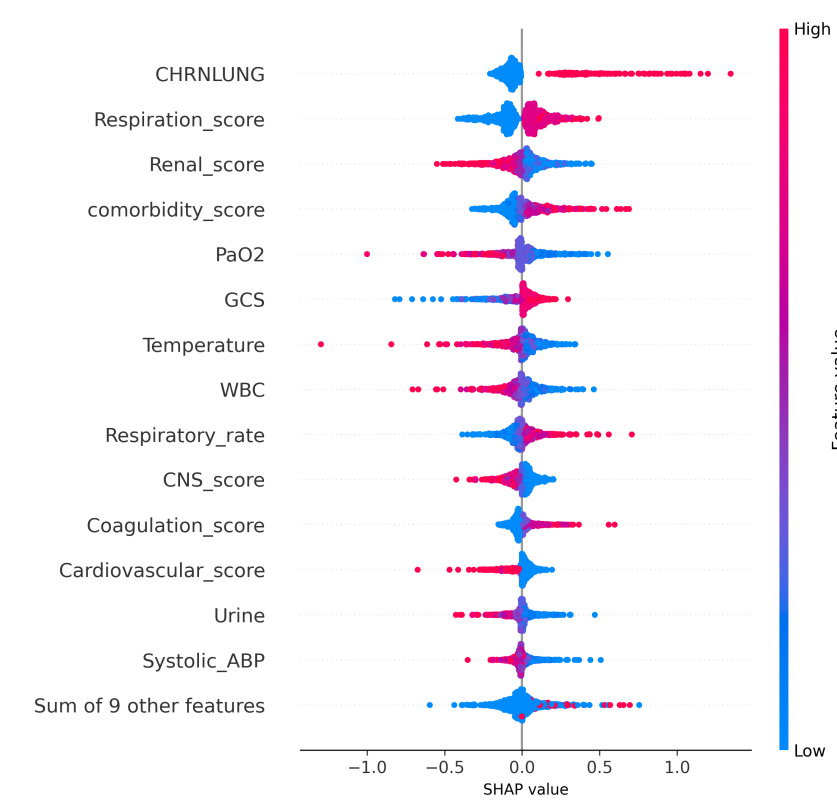

(c) Seed 3

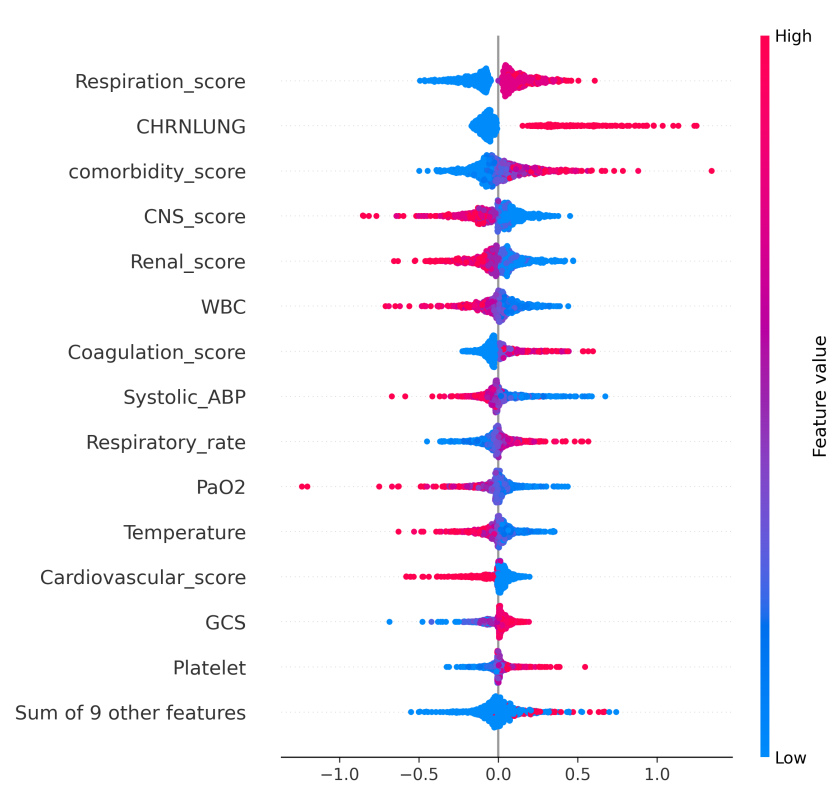

(d) Seed 4

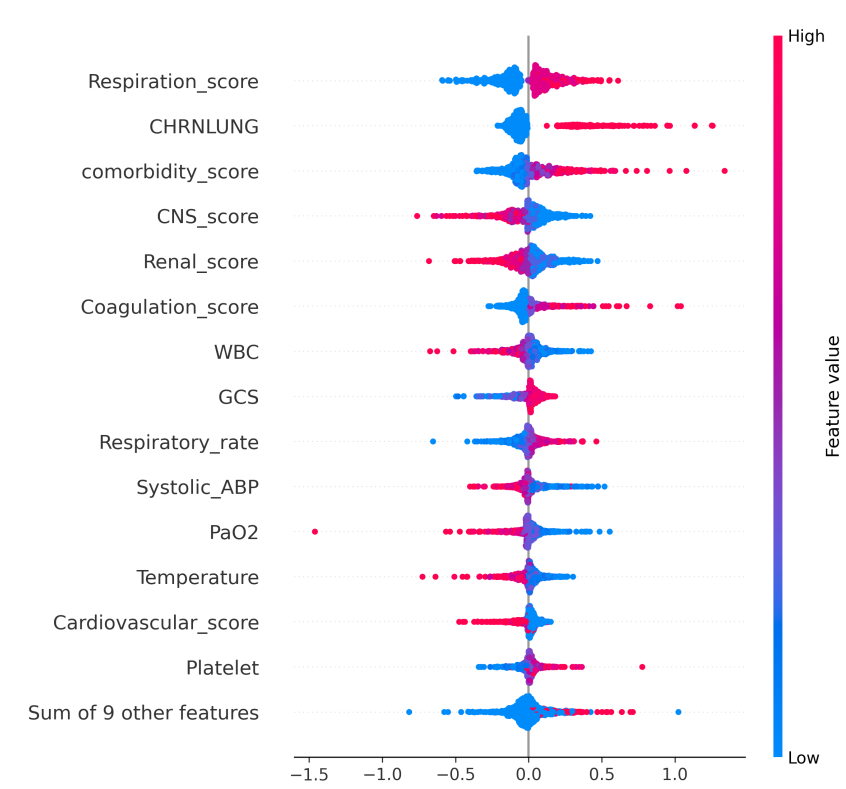

(e) Seed 5

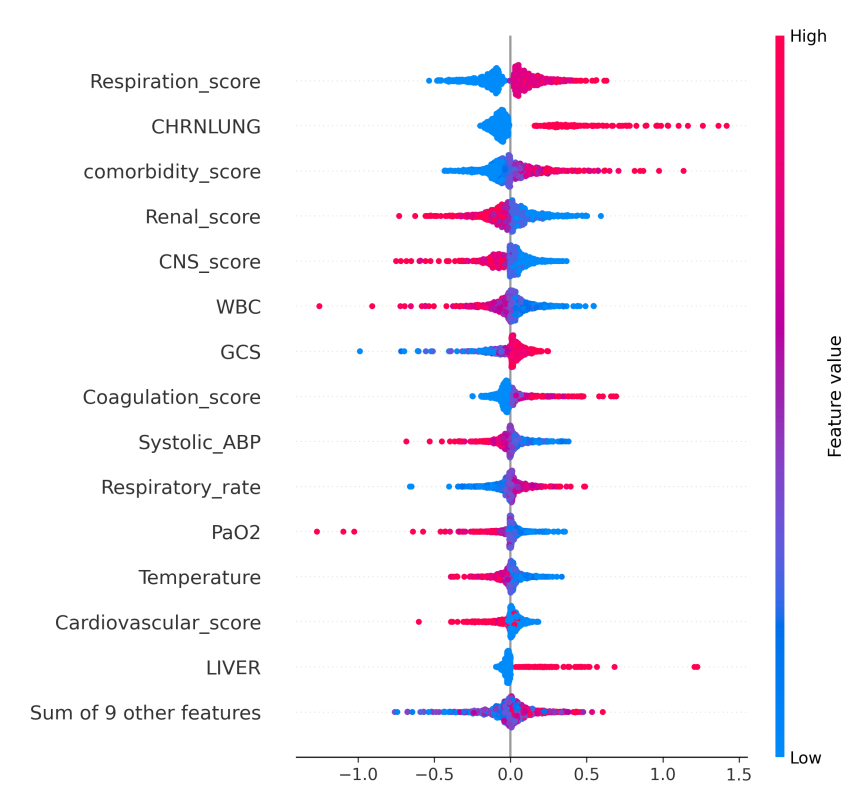

(f) Seed 6

Figure E.8: Model Stability across different initialization

That is, only patients with covariates > 0.05 at positive β_τ indices receive a positive effect; others receive none. This yields a positive subgroup comprising 68% of samples. Using this DGP, we test MOSIC with beta = 1e-5, alpha = 0, and c in {0.6, 0.7, 0.8}. The precision and recall of subgroup identification are evaluated. Results in Table F.1 are reported as mean ± standard deviation over 100 runs. Performance aligns with theory: precision/recall degrade when c (the group size constraint) exceeds/falls below the true subgroup size, reflecting constraint-driven trade-offs.

G Evaluation of Type I error

Although our primary focus is on multi-constraint subgroup identification rather than statistical inference, we also evaluate Type I error of the identified subgroup. We use a data-splitting approach to test whether identified subgroups arise spuriously under the null

c	ATE	Group Size	Precision	Recall
0.6	0.92±0.07	0.60±0.01	0.93±0.05	0.83±0.05
0.7	0.84±0.05	0.70±0.01	0.88±0.04	0.92±0.04
0.8	0.75±0.04	0.80±0.01	0.80±0.03	0.96±0.04

Table F.1: Performance of binary subgroup identification

c	ATE	Group Size	Type I error
0.4	-0.0011 ± 0.0401	0.3991 ± 0.0141	0.12
0.6	-0.0018 ± 0.0298	0.6020 ± 0.0141	0.00
0.8	-0.0000 ± 0.0212	0.8038 ± 0.0121	0.00

Table G.1: Type I error under different group size constraints.

hypothesis, where all individuals have zero treatment effect. Using data splitting, Type I error of the selected subgroup can be evaluated after we build the subgroup assignment model. Specifically:

(1) Split the dataset (e.g., 50-50) into training (subgroup selection) and holdout (inference);
(2) Train MOSIC on the training set to learn the subgroup model;
(3) Apply the model to the holdout set to identify the subgroup, then compute its subgroup ATE, denoted as ATE_{hold_out};
(4) Test the null hypothesis on the holdout set:
   (a) Construct the distribution of subgroup ATE under the null (here by directly sampling from the test distribution, with bootstrap subsample size equaling the target subgroup size specified by the parameter c);
   (b) Compare ATE_{hold_out} to this distribution, and determine whether the null hypothesis should be rejected.
(5) Repeat steps 1-4 on additional synthetic data instances, aggregate results, and estimate type-I error.

We generated 100 synthetic datasets using the same DGP in Appendix C.2 except that β_τ is set to $\mathbf{0}$. This aligns with the null hypothesis: all individuals have zero treatment effect. For each instance, we set the bootstrap iterations to 10000. Type I error rate is computed as the proportion of instances in which the null hypothesis is rejected at the 5% significance level.

As shown in Table G.1, the Type I error rate increases when the parameter c is small, consistent with theoretical expectations. Smaller subgroups lead to higher variance in ATE estimates, highlighting a fundamental trade-off between real-world constraints and statistical reliability.

H Runtime Analysis

Each method is run three times, and the average run time is reported; these experiments are run on CPU only to reflect clinical computing environments with limited resources. Since MOSIC's nuisance estimation step uses Dragonnet and Logistic regression, it shares the same nuisance-fitting cost as Dragonnet (Logistic Regression is negligible). Hence, the reported runtime for MOSIC reflects only the additional cost of the optimization step. As shown in Table 4, this overhead is small relative to nuisance estimation, indicating that computation is unlikely to be a deployment bottleneck.
